# Lifestyle shapes genome architecture and codon usage bias in *Staphylococcus aureus* bacteriophages, suggesting stronger host adaptation in their virulent lineages

**DOI:** 10.3389/fmicb.2026.1829197

**Published:** 2026-05-20

**Authors:** Ana Gamkrelidze, Davit Janelidze, Saba Kobakhidze, Tinatin Elbakidze, Mamuka Kotetishvili

**Affiliations:** School of Science and Technology, One Health Institute, University of Georgia, Tbilisi, Georgia

**Keywords:** bacteriophage, codon usage, GC content, lifestyle, prophage, temperate, virulent

## Abstract

Bacteriophages are major drivers of *Staphylococcus aureus* evolution, mediating predation, lysogeny, and the horizontal transfer of virulence and antibiotic-resistance genes. However, how lifestyle shapes their genomic architecture and synonymous codon usage remains poorly understood. To address this, we performed a comparative analysis of 30 experimentally verified virulent phages, 30 temperate phages, and 30 intact prophages from diverse *S. aureus* strains. We assessed genome heterogeneity and patterns of synonymous codon usage across these groups. Genome- and gene-size distributions were examined, along with gene-level metrics including overall and positional GC content, Codon Adaptation Index (CAI), and the effective number of codons (*Nc*). Virulent phages showed the highest genomic heterogeneity: nearly two-thirds of pairwise genome comparisons (59.1%) displayed complete divergence with no detectable similarity; their genomes also followed a strongly bimodal size distribution (median ≈130 kb; range 16.8 kb−148.5 kb). In contrast, temperate phages had smaller, tightly constrained genomes (median ≈43 kb; IQR 1.2 kb) and formed a continuous network of recombinational connectivity without extreme divergence. Prophages displayed intermediate characteristics, with larger and more variable genome sizes. The clearest lifestyle-associated signal appeared at the synonymous level. Genes from virulent phages had significantly lower GC content at third codon positions (%GC3), higher CAI, and lower *Nc* than genes from temperate phages and prophages (large Cliff's δ effects, Holm-adjusted *p* < 0.001). Importantly, these differences remained consistent across functional gene categories, including replication, structural, lysis, regulatory, and repair genes, and persisted after controlling for compositional bias. Together, these results reveal distinct evolutionary trajectories among *S. aureus* phages. The temperate phages appear to diversify through modular recombination within interconnected genomic networks, whereas the virulent phages show episodic divergence coupled with distinct codon usage patterns that may reflect enhanced translational selection, although partially influenced by compositional bias.

## 1 Introduction

Bacteriophages (phages) are ubiquitous viruses that profoundly influence bacterial evolution and ecology through predation and horizontal gene transfer. Within *Staphylococcus aureus* populations, phages frequently serve as vehicles for the transmission of virulence determinants and antibiotic-resistance genes among strains. For example, *S. aureus* isolates commonly harbor multiple prophages encoding factors such as Panton–Valentine leukocidin (PVL), enterotoxins, and immune-evasion proteins, suggesting that phage-mediated transduction represents a primary mechanism for the horizontal transfer of accessory genes (Xia and Wolz, 2014; Ingmer et al., 2019). Certain resident prophages also possess the ability to mobilize large genomic regions, including the virulence island νSaβ that encodes superantigens and leukotoxins (Moon et al., 2015). Even larger genetic transfers have been observed through lateral transduction, whereby phage-induced “piggybacking” by staphylococcal pathogenicity islands (SaPIs) enables the co-transfer of two pathogenicity islands (vSaα and vSaβ) within a single phage particle (Chee et al., 2023). Furthermore, experimental studies have demonstrated that phage lysates from multidrug-resistant *S. aureus* strains can transduce tetracycline resistance to susceptible strains (Mitsuhashi et al., 1965), and that phages induced from methicillin-resistant *S. aureus* (MRSA) ST398 are capable of packaging the entire *mecA*-containing SCCmecV element (Chlebowicz et al., 2014). However, the global genomic features associated with the lifestyles of *S. aureus* phages, particularly in the context of phage-host interactions that drive such evolution, remain poorly understood.

Phages generally exhibit two principal lifestyles: temperate (lysogenic) phages integrate into their host genomes and persist as prophages, whereas virulent (lytic) phages immediately replicate and lyse their host (Howard-Varona et al., 2017). These lifestyles impose distinct selective regimes on phage genomes (Prabhakaran et al., 2015; Kobakhidze et al., 2025). Temperate phages experience prolonged association with their hosts and may evolve genomic features that better match host codon usage patterns, whereas virulent phages are often subject to selection favoring rapid gene expression and efficient replication. Consequently, lifestyle differences may influence phage genome architecture and codon usage patterns. Recent work on *Listeria monocytogenes* phages demonstrated that temperate phages tend to have smaller genomes and exhibit higher codon adaptation index (CAI) values than virulent phages, whereas virulent phages demonstrate stronger overall codon usage bias (Kobakhidze et al., 2025). In addition, staphylococcal phages are known to possess highly mosaic genomes with extensive gene flux among clusters (Oliveira et al., 2019), although it remains unclear how phage lifestyle influences the genomic codon usage metrics underlying this mosaicism. Previous studies have also shown that host identity, lifestyle, and phage taxonomy can shape codon usage bias in phage genomes (Ge et al., 2020), suggesting that lifestyle may play a role in translational optimization. Moreover, analyses across diverse phage systems have revealed that structural genes, particularly those encoding head and tail proteins, often display codon usage patterns strongly adapted to host preferences (Lucks et al., 2008), highlighting the importance of gene function in shaping codon usage evolution. Consistent with this view, comparative analyses of coliphages and staphylococcal phages have suggested that phage lifestyle modulates selective pressures on translational efficiency (Prabhakaran et al., 2015).

Nevertheless, several fundamental questions remain unresolved, at least with respect to staphylococcal phages. In particular, it remains unclear whether distinct *S. aureus* phage lifestyles are consistently associated with differences in genome architecture, gene-length distributions, and codon usage. Likewise, the extent to which lifestyle-related selective regimes shape genome-wide compositional properties, such as overall GC content, position-specific GC variation, and metrics of codon adaptation and codon bias, remains largely unexplored in *S. aureus* phages. Addressing these questions requires large-scale comparative analyses that integrate genome similarity, compositional features, and gene-level functional organization across diverse phage groups.

In this study, we performed a comprehensive comparative analysis of virulent and temperate phages, together with prophage genomes associated with *S. aureus*, to investigate lifestyle-associated patterns of genome evolution and codon usage. Using a balanced dataset of phage and prophage genomes, we examined genome heterogeneity, genome size variation, and gene-level compositional properties, CAI, effective number of codons (*Nc*), and position-specific GC content. We further evaluated multivariate compositional differences among phage lifestyles and assessed how codon usage patterns vary across functional gene categories. By integrating genome-scale and gene-level analyses, this study aims to clarify how phage lifestyle shapes genome architecture and translational strategies in *S. aureus* phages, thereby providing new insights into phage-host coevolution and the evolutionary constraints governing phage genomic organization.

## 2 Materials and methods

### 2.1 Selection of phage and prophage genomes

The complete genome sequences of *Staphylococcus aureus* phages were retrieved from the NCBI GenBank nucleotide database. The selection analyses included publicly available complete genomes from the classified virulent and temperate phages, as well as from the prophages of *S. aureus* strains. Only phages with experimentally verified lifestyles, previously determined under laboratory conditions and reported in peer-reviewed publications, were included.

Intact prophages were identified using PHASTER (PHAge Search Tool – Enhanced Release) (Arndt et al., 2016; Zhou et al., 2011) across the complete genomes of different *S. aureus* strains obtained from GenBank. Information on the isolation sources of these strains, along with their GenBank accession numbers, is provided in Supplementary Table S1. Although the *S. aureus* strains were randomly selected, only the genomes of intact and genetically diverse prophages, as identified by PHASTER and their comparative analyses, respectively, were included in this study. We aimed to include in the study an equal number of genomes for each phage lifestyle group to adhere to a balanced design (30 genomes per lifestyle group), prioritizing genetic diversity within each set. To determine the systematic affiliation of the phages, we compared their genomes against the curated INPHARED reference dataset (Cook et al., 2021), and used taxMyPhage (Millard et al., 2025) for automated ICTV-aligned taxonomic assignment, allowing database-based contextualization together with formal genus/species classification.

To ensure within-group heterogeneity as one of the inclusion criteria for constructing final datasets per each lifestyle group, pairwise genome comparisons were performed for each initial subset (*n*[*n* – 1]/2). Within each lifestyle group, whole-genome nucleotide sequences were subjected to all-versus-all BLAST comparisons. For each genome pair, two similarity metrics were extracted: percent DNA identity *(*%ID) and query coverage (%Cov). Because BLAST comparisons are directional (genome A versus genome B, and vice versa), reciprocal values were averaged to obtain a single metric for each unordered genome pair. Comparisons yielding no significant similarity (NSS) under the uniform BLAST parameters were retained for the downstream analyses and were not treated as missing data.

To quantify genomic heterogeneity using an integrated metric, similarity values were transformed into distance components:


$$\begin{array}{l} d_{id}=1-\frac{\%ID}{100}\\ d_{cov}=1-\frac{\%Cov}{100} \end{array}$$

A combined genomic distance (CGD) was then calculated as:


$$\begin{array}{l} d=\sqrt{d_{id}^{2}+d_{cov}^{2}} \end{array}$$

This metric incorporated both nucleotide divergence and the genomic alignment breadth with the theoretical range of the metric extending from 0 (100% identity across 100% coverage) to:


$$\begin{array}{l} d_{\max}=\sqrt{{\left(1-0\right)}^{2}+{\left(1-0\right)}^{2}}=\sqrt{2}\approx 1.414 \end{array}$$

Comparisons classified as NSS represented the absence of detectable similarity under consistent alignment criteria, and therefore, reflected extreme genomic divergence—rather than missing observations. For the analyses incorporating all pairwise comparisons, NSS pairs were assigned the theoretical maximum value of the combined distance metric:


$$\begin{array}{l} d_{NSS}=\sqrt{2} \end{array}$$

This assignment placed NSS at the mathematical upper bound of the defined metric, ensuring internal consistency and enabling direct comparison of heterogeneity across lifestyle groups.

Two complementary summary approaches were further employed. Specifically, for genome pairs yielding measurable %ID- and %Cov-values, the following descriptive statistics were calculated for each lifestyle group: mean, median, standard deviation (SD), and interquartile range (Q1–Q3) for %ID, %Cov, and CGD. This analysis allowed us to characterize genomic heterogeneity among genome pairs sharing detectable similarity within each lifestyle group. Then, the derived CGD-values were summarized across all possible genome pairs, with NSS assigned $d=\sqrt{2}$; the following metrics were calculated: a total number of pairwise comparisons, a number and proportion of valid similarity pairs, a number and proportion of NSS pairs, mean CGD, median CGD, SD, Q1–Q3, and maximum observed distance. This dual framework allowed the quantification of overall within-group genomic heterogeneity, while explicitly incorporating extreme divergence events. Because NSS represented informative absence of detectable similarity rather than random missingness, heterogeneity was evaluated both among detectable similarities alone and across all possible genome pairs.

Assigning maximum distance to genome pairs with NSS can overestimate evolutionary divergence, as homologous relationships below BLAST detection thresholds may remain undetected; however, in this study, this approach, combined with BLAST-derived sequence identity and query coverage estimates, and considered in the context of the highly mosaic nature of phage genomes, was used solely to ensure the selection of genetically diverse genomes rather than to infer precise evolutionary relationships, and is therefore highly unlikely to affect downstream codon usage analyses.

### 2.2 Comparative analysis of genome-size variation across phage lifestyles

Genome sizes (bp) were analyzed for three bacteriophage lifestyle categories: temperate, prophage, and virulent (*n* = 30 per group; total *N* = 90). For each group, descriptive statistics were calculated, including mean, sample standard deviation (SD), median, minimum, maximum, and empirical percentiles (P10, P25 [Q1], P50, P75 [Q3], P90). The interquartile range (IQR = Q3–Q1) was used as a robust measure of dispersion. Because genome-size distributions may deviate from normality and exhibit skewness or multimodality, distribution-aware statistics (median, IQR, and percentiles) were emphasized alongside mean and SD. Differences among lifestyle groups were assessed using the Kruskal–Wallis *H* test, a rank-based non-parametric alternative to one-way ANOVA appropriate for non-normally distributed data and unequal variances (Kruskal and Wallis, 1952). All observations (*N* = 90) were pooled and converted to ranks, with ties assigned average ranks. The H statistic was evaluated against a chi-square distribution with *k*−1 degrees of freedom (*k* = 3). When the omnibus test was significant, pairwise comparisons were performed using Dunn's *post-hoc* test based on differences in mean ranks (Dunn, 1964). To control the family-wise error rate across three comparisons, Bonferroni and Holm adjustments were applied, with Holm-adjusted *p-*values used for primary inference (Holm, 1979). Effect sizes for the Kruskal–Wallis test were calculated using rank-based eta-squared (η^2^) and epsilon-squared (ε^2^), which estimate the proportion of variance in rank ordering attributable to lifestyle category (Tomczak and Tomczak, 2014). All tests were two-sided with a significance threshold of α = 0.05.

To quantify the magnitude of group differences determined by the Kruskal–Wallis test, rank-based effect size measures were calculated. While the Kruskal–Wallis test evaluates whether statistically significant differences exist among groups, effect size metrics estimate the proportion of variability attributable to group membership. For non-parametric group comparisons, eta-squared (η^2^) and epsilon-squared (ε^2^) were considered to be estimators derived from the Kruskal–Wallis *H* statistic (Tomczak and Tomczak, 2014; Kerby, 2014).

Effect sizes were computed using the following formulas:


$$\begin{array}{l} \eta^{2}=\frac{H-k+1}{N-k}\\ \varepsilon^{2}=\frac{H-k+1}{N-1} \end{array}$$

where *H* denotes the Kruskal–Wallis test statistic, *k* is the number of groups, and *N* is the total sample size.

### 2.3 Assessment of genome-wide codon usage across phage lifestyle groups

Protein-coding genes from the genomes of *S. aureus* virulent and temperate phages, together with those from selected *S. aureus* prophages, were analyzed to assess genome-wide codon usage patterns across phage lifestyle groups. Specifically, GC content (%GC), position-specific GC content (%GC1, %GC2, %GC3), Codon Adaptation Index (CAI), and the effective number of codons (*Nc*) were calculated for each gene from the virulent and temperate phages, and prophages, using the CAIcal server (http://genomes.urv.es/CAIcal/). Codon usage frequencies were calculated based on a total of 1,517,117 codons from 4,964 protein-coding sequences (CDSs) of *Staphylococcus* aureus subsp. aureus strain MW2, as provided in the Kazusa Codon Usage Database (https://www.kazusa.or.jp/codon/cgi-bin/showcodon.cgi?species=196620). CAI values were calculated according to Sharp and Li (1987), and *Nc* values were computed following Wright (1990).

Gene-level sequence metrics were calculated for all CDSs, including CAI, *Nc*, overall and position-specific GC content, and gene length (*nt*) across all three phage lifestyle categories. Because the distributions of these variables deviated from normality (assessed by visual inspection and Shapiro–Wilk tests), nonparametric statistical methods were used. Global differences among the three lifestyle groups were evaluated using the Kruskal–Wallis rank-sum test (Kruskal and Wallis, 1952). Effect sizes for the Kruskal–Wallis tests were estimated using epsilon-squared (ε^2^), representing the proportion of variance explained by lifestyle. Effect sizes were interpreted as negligible (< 0.01), small (0.01–0.08), moderate (0.08–0.26), or large (>0.26), following the commonly used guidelines (Tomczak and Tomczak, 2014).

For metrics exhibiting significant global differences (*p* < 0.05), pairwise comparisons between the lifestyle groups were performed using the Mann–Whitney *U* test (Wilcoxon rank-sum test) (Mann and Whitney, 1947). To control the family-wise error rate associated with multiple testing, *p*-values were adjusted using the Holm–Bonferroni procedure. Holm-adjusted *p*-values (*p*_adj_) < 0.05 were considered statistically significant (Holm, 1979). Effect sizes for pairwise contrasts were calculated using Cliff's delta (δ), a nonparametric measure of stochastic dominance that estimates the probability that a randomly selected observation from one group exceeds a randomly selected observation from another group. Cliff's δ values were interpreted as negligible (|δ| < 0.147), small (0.147–0.33), moderate (0.33–0.474), or large (≥0.474) (Cliff, 1993). Direction of differences between groups was reported explicitly (e.g., Temperate > Virulent) to facilitate biological interpretation.

We further investigated whether *S. aureus* virulent phages exhibiting larger genome sizes and higher CAI values also encode a greater number of tRNAs. The number of tRNAs per genome was determined by counting annotated tRNA entries, and a binary variable for tRNA presence (presence/absence) was defined based on whether at least one tRNA was detected. Associations between the variables were assessed using Spearman's rank correlation coefficient to account for non-normal distributions. Specifically, correlations were tested between genome size and tRNA count, and between CAI and tRNA presence. All statistical analyses were performed in Python using the *pandas* and *scipy* libraries.

### 2.4 Pairwise multivariate comparisons among phage lifestyles

Pairwise differences in overall gene-level compositional profiles among phage lifestyles were assessed, using permutational multivariate analysis of variance (PERMANOVA). PERMANOVA is a non-parametric analog of multivariate analysis of variance that does not assume multivariate normality (Anderson, 2001). For each lifestyle group, every gene was represented by a vector of sequence metrics including *nt*, CAI, *Nc*, overall %GC, %GC1, %GC2, and %GC3. Prior to the above analysis, all variables were standardized using *z*-score transformation to remove scale differences among metrics. Euclidean distances were computed from the standardized data to quantify multivariate dissimilarity among genes. Because of the large dataset size, each pairwise comparison was performed on a random subset of genes (*N* = 1,953 per comparison) corresponding to the size of the smallest dataset, to ensure computational feasibility while maintaining statistical power. PERMANOVA was implemented using the adonis2 function in the *vegan R* package. Statistical significance was evaluated using permutation testing with 999 permutations, in which lifestyle labels were randomly reassigned among observations to generate a null distribution of the pseudo-*F* statistic. The proportion of multivariate variance explained by group membership was quantified, using the coefficient of determination (*R*^2^). Differences in multivariate dispersion among lifestyles were additionally evaluated using permutational analysis of multivariate dispersions (PERMDISP).

### 2.5 Spearman and partial correlation analyses of gene-level codon usage variables

The subsequent analyses were performed to determine if there was statistically meaningful correlation(s) among *nt*, overall %GC, %GC1, %GC2, %GC3, CAI, and *Nc* variables genome-wide separately within per phage lifestyle. For these analyses, *nt* estimates were log10-transformed to reduce right-skewness and stabilize variance. Monotonic associations among *nt*, CAI, *Nc*, overall %GC, %GC1, %GC2, %GC3 estimates were quantified using Spearman's rank correlation coefficient (ρ) (Spearman, 1904). Two-sided significance tests were performed under the null hypothesis ρ = 0. Because multiple correlations were evaluated within each lifestyle, *p*-values were adjusted using the Benjamini–Hochberg false discovery rate (FDR) procedure (Benjamini and Hochberg, 1995), and adjusted *p*-values are reported as FDR-adjusted *p*-values. Ninety-five percent confidence intervals for Spearman correlation coefficients were estimated using bootstrap resampling (1,000 iterations) with replacement within each lifestyle (Efron and Tibshirani, 1993).

To evaluate whether observed associations were independent of nucleotide compositional bias, partial Spearman correlations were computed controlling for both %GC3 and overall %GC. Partial correlations were calculated using a rank-based residualization approach: variables were rank-transformed, residuals were obtained from linear regression of ranked variables on ranked covariates, and Spearman correlation was computed between residuals (Fisher, 1924; Kendall and Stuart, 1979). FDR correction was applied to partial correlation *p*-values as described above. Effect sizes (ρ) and confidence intervals were interpreted alongside adjusted *p*-values to distinguish statistical significance from biological relevance.

### 2.6 Statistical comparative analyses of functional gene groups within and among phage lifestyles

We analyzed and compared *nt*, overall %GC, %GC1, %GC2, %GC3, CAI, and *Nc* patterns across the well-annotated structural, regulatory, lysis, replication, and repair genes among phage lifestyles (virulent, temperate, and prophage). Structural genes included those involved in DNA packaging, head morphogenesis and maturation, portal-neck-head-tail assembly, tail morphogenesis, and host adsorption and penetration. Regulatory genes comprised repressors and antirepressors controlling the lysogenic-lytic switch, together with transcriptional activators, DNA-binding transcriptional regulators, sigma and anti-sigma factors, two-component response regulators, and recombination-associated regulators. The domain architecture of proteins encoded by regulatory genes with unusually long nucleotide sequences (3,459 *nt*) was analyzed using InterPro v108.0 with InterProScan v5.77 and SWISS-Model (https://swissmodel.expasy.org/) to evaluate whether they represent fusion proteins or potential annotation artifacts in the GenBank database. Lysis genes included those encoding for endolysins (amidases and lysin motif-containing proteins), membrane-permeabilizing holins and related lysis proteins, and additional cytolytic proteins such as hemolysin-family proteins. Replication genes encoded for replication initiation proteins, DNA polymerases with proofreading exonucleases, helicases and helicase-loading proteins, primases and primase-helicase complexes, RNA polymerase subunits, and replication-repair nucleases. Repair genes included those involved in DNA methylation and epigenetic modification, restriction-modification system inhibition (e.g., ArdA and Lar proteins), and other nucleotide-modifying enzymes such as dCMP hydroxymethylase, DNA sulfur modification protein DndB, and RNA methyltransferases.

The genome-level medians of *nt*, overall %GC, %GC1, %GC2, %GC3, CAI, and *Nc* were analyzed and compared for each functional gene category within and among three phage lifestyles (temperate, virulent, and prophage). Because data distributions deviated from normality and homogeneity of variance, nonparametric methods were applied throughout. To assess the main effects of functional category and lifestyle, as well as their interaction, a Scheirer–Ray–Hare test was performed, representing a nonparametric extension of two-way ANOVA based on ranked data (Scheirer et al., 1976). Effect sizes for omnibus tests were estimated using epsilon-squared (ε^2^) quantifying the proportion of variance explained (Tomczak and Tomczak, 2014). Within each lifestyle group, differences among functional gene categories were evaluated using the Kruskal–Wallis rank-sum test (Kruskal and Wallis, 1952). When significant, pairwise comparisons were conducted using Dunn's rank-based multiple comparison test (Dunn, 1964). To control the family-wise error rate, *p*-values were adjusted using the Holm sequential correction procedure (Holm, 1979).

To evaluate lifestyle-associated differences within each functional gene category, Kruskal–Wallis tests were applied across the three lifestyle groups (Kruskal and Wallis, 1952). Significant results were followed by pairwise Mann–Whitney *U* tests (two-tailed) (Mann and Whitney, 1947), equivalent to Dunn's procedure for three-group comparisons. Holm-adjusted *p*-values < 0.05 were considered statistically significant. Pairwise effect sizes were quantified using Cliff's delta (δ), a nonparametric measure of stochastic dominance between two independent groups (Cliff, 1993). Effect magnitudes were interpreted according to conventional thresholds (|δ| ≥ 0.147, small; ≥0.330, medium; ≥0.474, large; ≥0.800, very large), with δ indicating the direction of dominance. All tests were two-tailed, and adjusted *p*-values < 0.05 were considered statistically significant.

Dataset construction and BLAST output processing were implemented in Python v3.14 (pandas v3.0.1, NumPy v2.4.2, scipy v1.18). Inferential statistical analyses were conducted in R v4.5.2 (readxl v1.4.5, vegan v2.7.3, dplyr v1.2.0, effsize v0.8.1), where group differences were evaluated using the Kruskal–Wallis test followed by Dunn's test with multiple-testing correction.

## 3 Results

### 3.1 Genome heterogeneity across phage lifestyles

A total of 30 genomes per phage lifestyle group were selected from the NCBI GenBank database for analysis. The selected virulent and temperate phages belonged collectively to the families *Herelleviridae* and *Rountreeviridae*, and the subfamilies *Azeredovirinae* and *Bronfenbrennervirinae* (Supplementary Table S2). As shown, the virulent group comprised multiple morphotypes, including myovirus-like, siphovirus-like, and podovirus-like phages, whereas the temperate group was predominantly composed of siphovirus-like phages. Intact prophages were identified using PHASTER across the genomes of diverse *S. aureus* strains (Supplementary Table S1). As shown, these prophages were systematically affiliated with the phages that infect at least *S. aureus*. Among genome pairs with detectable similarity, the virulent phages show the highest mean DNA identity (86.89%), the temperate phages the lowest (83.51%), and the prophages intermediate values (84.62%) (Supplementary Table S3). Genome coverage exhibit broad dispersion across all groups, with low median values (18.5%−23.0%), indicating that most similarities were confined to partial genomic regions. When %ID and %Cov were integrated into the CGD metric, the temperate phages displayed the greatest divergence among valid alignments (mean distance 0.793; median 0.936), followed by the prophages (mean 0.735; median 0.812), whereas the virulent phages showed the lowest mean distance (mean 0.586; median 0.793).

When all possible genome pairs were incorporated into the within-group heterogeneity analyses, marked differences emerged in the frequency of complete divergence events (Supplementary Table S4). The virulent phages exhibited a very high proportion of NSS comparisons (59.1%), whereas the prophages showed relatively few (5.5%), and the temperate phages none. After assigning NSS pairs the theoretical maximum distance, the virulent phages displayed the highest overall heterogeneity (mean combined distance 0.942; median 1.414), followed by the prophages (mean 0.815; median 0.916) and the temperate phages (mean 0.744; median 0.861). Thus, the virulent phages showed the greatest overall heterogeneity due to a very high frequency of complete divergence events, with more than half of genome pairs lacking detectable similarity. The temperate phages exhibited pronounced divergence among detectable alignments but lacked extreme dissimilarity, while the prophages demonstrated intermediate heterogeneity with limited maximal divergence. Overall, all three lifestyle groups showed substantial genomic heterogeneity, although the magnitude and pattern of divergence differed among the virulent, temperate, and prophage populations examined.

In the analysis, we also investigated separately as an outlier the *S. aureus* phage SA1 (Supplementary Table S2) with the genome size of 260,727 bp, being classified as a jumbo phage. The above phage was previously validated to be a virulent phage under the respective wet-laboratory experiments (Zhang et al., 2022). The BLAST analysis revealed NSS patterns with any virulent phage from our virulent phage collections examined. It should be indicated that no other jumbo phages met the above selection criteria, including experimentally validated lifestyle, among those with the genome sequences available in the publicly accessible DNA sequence databases.

### 3.2 Lifestyle-associated differences in phage genome size

Genome sizes differed markedly among the temperate and virulent phages, and the prophages (Table 1). The Temperate phages exhibited a highly constrained genome-size distribution, with a median of 43,162 bp and a narrow interquartile range (IQR = 1,228 bp). The 10th−90th percentile range (41,834 bp−45,311 bp) further confirmed limited dispersion, and the values spanned only ~6 kb overall (39,968 bp−45,985 bp). The prophages displayed substantially larger and more variable genomes. The median genome size was 59,638 bp, approximately 16 kb larger than that of the temperate phages. Dispersion was markedly increased (IQR = 12,875 bp), and the full range extended from 43,363 bp to 130,595 bp. The percentile values showed a consistent upward shift relative to the temperate phages across the entire distribution (P10–P90). The virulent phages exhibited the greatest genomic heterogeneity: although their mean genome size was 85,337 bp, the median was considerably higher (130,493 bp), reflecting a strongly asymmetric and likely bimodal distribution. The lower percentiles (P10 = 17,150 bp; P25 = 17,808 bp) indicated a cluster of small genomes (~17 kb−18 kb), whereas the upper percentiles (P75 = 140,732 bp; P90 = 142,363 bp) reflected a second regime of the large genomes (~130 kb−140 kb). The resulting IQR (122,924 bp) was an order of magnitude larger than that observed in the prophages and nearly 100-fold greater than in the temperate phages.

**Table 1:** Genome size (bp) descriptive statistics (mean ± SD, median, 10th−90th percentiles, interquartile range IQR, minimum–maximum) for the *S. aureus* temperate and virulent phages, and for this species prophages.

Group	n	Mean	SD	Median	P10	P25	P50	P75	IQR	P90	Min	Max
Temperate	30	43,297	1,423	43,162	41,834	42,738	43,162	43,966	1,228	45,311	39,968	45,985
Prophage	30	61,021	15,655	59,638	48,163	51,451	59,638	64,326	12,875	72,222	43,363	130,595
Virulent	30	85,337	59,906	130,493	17,150	17,808	130,493	140,732	122,924	142,363	16,784	148,511

Pairwise comparisons using Dunn's *post-hoc* test (Table 2) revealed significant differences in mean ranks between the temperate phages and the prophages (Holm-adjusted *p* = 1.56 × 10^−5^), and between the temperate and virulent phages (Holm-adjusted *p* = 0.004). In both comparisons, the temperate phages had substantially lower mean ranks (28.267) than the prophages (59.000) and the virulent (49.233) phages, indicating consistently smaller genome sizes. In contrast, the difference between the prophages and virulent phage was not statistically significant after Holm correction (*p* = 0.148), despite the prophages exhibiting a higher mean rank (59.000 vs. 49.233). The absence of statistical significance likely reflects the pronounced bimodality and extreme dispersion within the virulent group, which reduces power in rank-based comparisons of central tendency. The Kruskal–Wallis test revealed significant differences in genome size distributions among the three phage lifestyle groups. To assess the magnitude of this effect, rank-based effect size estimates were calculated. Using the observed test statistic (*H* = 21.678225; *N* = 90; *k* = 3), eta-squared was estimated at η^2^ = 0.226 and epsilon-squared at ε^2^ = 0.221. The epsilon-squared value of ~0.22 indicated that roughly 22% of the variability in ranked genome size was attributable to phage lifestyle classification. Overall, genome-size variation across phage lifestyles was characterized by (i) highly constrained genomes in the temperate phages, (ii) intermediate but expanded distributions in the prophages, and (iii) extreme heterogeneity and bimodality in the virulent phages.

**Table 2:** The results of Dunn's nonparametric *post-hoc* pairwise comparisons (including *z*-statistics, raw *p*-values, Bonferroni-corrected *p*-values, and Holm-corrected *p*-values) for observed differences in genome size across the *S. aureus* temperate and virulent phages, as well as this species prophages.

Comparison	Mean rank (G1)	Mean rank (G2)	*z*	*P* (raw)	*P* (Bonferroni)	*P* (Holm)
Temperate vs prophage	28.267	59	−4.556	5.21E-06	1.56E-05	1.56E-05
Temperate vs virulent	28.267	49.233	−3.108	0.002	0.006	0.004
Prophage vs virulent	59	49.233	1.448	0.148	0.443	0.148

To further investigate the observed bimodal genome size distribution among virulent phages, we examined whether their taxonomic affiliations were associated with the genome size clusters. Importantly, it was revealed that the two size clusters correspond closely to distinct viral families: the phages within the smaller genome cluster (~17 kb−18 kb) were found to belong the *Rountreeviridae* family, while the phages within the larger genome cluster (~130 kb−140 kb), in contrast, belonged to the *Herelleviridae* family.

### 3.3 Global comparisons and pairwise contrasts among phage lifestyles

A total of 3,760 virulent phage genes, 1,953 temperate phage genes, and 2,488 prophage genes were analyzed. Descriptive statistics revealed both shared characteristics and clear lifestyle-associated differences across gene length (*nt*), codon usage, and GC-related metrics (Table 3). Mean gene length was comparable among lifestyles (virulent: 604.37 *nt*; temperate: 606.67 *nt*; prophage: 637.82 *nt*). However, the *nt* patterns for the prophage genes exhibited slightly greater dispersion (SD = 682.32; IQR = 531) relative to those of the virulent (SD = 588.94; IQR = 474) and temperate (SD = 656.93; IQR = 495) phage genes. Median gene lengths were similar across groups (378 *nt*−405 *nt*), although the prophages genes exhibited the highest maximum value (8,274 *nt*), suggesting a greater representation of longer coding sequences in this group.

**Table 3:** The comparative genome-wide descriptive *nt*, CAI, *Nc*, and %GC (overall and codon-positional) statistics computed for all genes of the *S. aureus* virulent and temperate phages, and for those of this species prophages.

Metric	Lifestyle	Mean	SD	Min	Max	P10	P25	P50	P75	P90	IQR
*Nt*	Virulent	604.37	588.94	63	6,225	183	258	399	732	1,272	474
	Temperate	606.67	656.93	90	6,225	177	243	378	738	1,368	495
	Prophage	637.82	682.32	75	8,274	174	249	405	780	1,301.7	531
CAI	Virulent	0.723	0.051	0.494	0.884	0.663	0.696	0.726	0.753	0.787	0.057
	Temperate	0.653	0.053	0.459	0.828	0.585	0.619	0.655	0.687	0.721	0.068
	Prophage	0.674	0.058	0.449	0.865	0.598	0.633	0.677	0.713	0.748	0.080
%GC	Virulent	29.772	3.556	14.9	42.3	25.5	27.6	29.6	32	34.51	4.4
	Temperate	33.764	3.425	22	45	29.2	31.5	33.8	36.1	38.3	4.6
	Prophage	32.882	3.541	20.500	44.900	28.200	30.700	33.000	35.200	37.500	4.500
%GC1	Virulent	41.249	6.585	6.8	59.2	33.3	37.2	41.75	45.325	49	8.125
	Temperate	43.393	5.853	13.6	61.9	36	39.6	44	47	50.58	7.4
	Prophage	42.884	6.117	18.2	60.2	35.2	39	43.3	46.9	50	7.9
%GC2	Virulent	27.743	6.107	6.5	57.3	20.59	23.475	27.5	31.4	35.8	7.925
	Temperate	29.348	5.762	13.8	53	22.4	25.5	29.3	33.1	37	7.6
	Prophage	29.287	5.560	12	50.9	22.4	25.6	29.2	33.2	36	7.6
%GC3	Virulent	20.323	5.168	3	51.9	14.4	17.3	19.8	22.6	26.8	5.3
	Temperate	28.551	5.948	14.9	52.3	21	24.3	28	32.5	36.5	8.2
	Prophage	26.473	6.122	10.2	53.2	19.1	22.2	25.8	30.6	34.8	8.4
*Nc*	Virulent	37.092	5.166	20	61	31.3	34.1	36.6	39.7	43.6	5.6
	Temperate	44.301	6.818	21.5	61	36.2	40.3	43.9	47.9	52.6	7.6
	Prophage	42.784	6.665	23.6	61	34.8	38.6	42.6	46.6	51.4	8

The CDSs from the virulent phage genomes showed the highest mean CAI (0.723 ± 0.051), followed by those from the genomes of the prophage (0.674 ± 0.058) and temperate phage (0.653 ± 0.053). The median CAI values illuminated the same trend (virulent: 0.726; prophage: 0.677; temperate: 0.655). The narrower IQR pattern was observed for the virulent phage genomes (0.057), compared with those of the temperate phage (0.068) and prophages (0.080).

Furthermore, genome size was analyzed as a continuous variable (in base pairs) to assess its association with tRNA content in virulent phages. A moderate positive correlation was observed between genome size and tRNA count (Spearman's ρ = 0.56, *p* = 0.0013), indicating that larger virulent phage genomes tend to encode a greater number of tRNAs. Besides, tRNA carriage was found to be strongly associated with the bimodal genome size distribution in these phages. Specifically, tRNAs were detected exclusively in the virulent phages (*n* = 9) with genome sizes ranging from 127.188 kb to 148.511 kb, although not all the phages within this size range (*n* = 16 in total) encoded tRNAs. In contrast, no significant relationship was detected between tRNA presence and CAI (Spearman's ρ = −0.07, *p* = 0.72).

The temperate phage genomes exhibited the highest mean GC content (33.76%), followed by those of the prophages (32.88%), whereas the virulent phage genomes had substantially lower GC content (29.77%). The median values closely reflected the means, and IQRs were similar across lifestyles (~4.4%−4.6%), indicating comparable variability despite differences in central tendency. Differences in GC content were most pronounced at the third codon position (%GC3). The temperate phages showed the highest mean %GC3 (28.55%), followed by the prophages (26.47%), while the virulent phages had markedly lower values (20.32%). The wider IQRs observed for the temperate (8.2) and prophage genomes (8.4), compared with virulent genomes (5.3), indicated greater variability in synonymous site composition across these groups. In contrast, %GC1 and %GC2 were more conserved across lifestyles: the temperate phages exhibited slightly higher mean %GC1 (43.39%) and %GC2 (29.35%) than the prophages (42.88% and 29.29%) and the virulent phages (41.25% and 27.74%), although these differences were less pronounced than those observed at GC3.

The CDCs of the virulent phage genomes exhibited the lowest mean *Nc* (37.09 ± 5.17), indicating stronger codon usage bias relative to those of the temperate phages (44.30 ± 6.82) and prophages (42.78 ± 6.67). The median *Nc* values followed the same pattern (36.6, 43.9, and 42.6, respectively). The narrower IQR for the virulent phage genomes (5.6), compared with those of the temperate phages (7.6) and prophages (8.0), further supports more constrained codon usage among the virulent phages.

The nonparametric Kruskal–Wallis tests indicated highly significant global differences for all the examined metrics except the *nt* patterns (Table 4): the strongest lifestyle effect was observed at %GC3, which exhibited a large effect size (ε^2^ = 0.324; *H* = 2658.519; *p* < 0.001), indicating that approximately one-third of the variance in GC3 among genes can be attributed to lifestyle differences. Substantial effects were also detected for CAI (ε^2^ = 0.257), *Nc* (ε^2^ = 0.251), and the overall GC content (ε^2^ = 0.212). In contrast, the GC content at the first and second codon positions showed only small effect sizes (%GC1 ε^2^ = 0.022; %GC2 ε^2^ = 0.021), despite highly significant *p*-values, indicating that these positions are comparatively constrained across lifestyles. However, the derived *nt*-values exhibited a negligible effect (ε^2^ = 0.001; *p* = 0.017).

**Table 4:** The Kruskal–Wallis test results for lifestyle-associated differences in codon usage bias (CAI, *Nc*) and nucleotide composition (overall %GC, positional %GC) across all genes of the *S. aureus* virulent and temperate phages, and those of prophages of this species.

Metric	*H*	*p*	ε^2^	Effect
CAI	2,112.487	0.00e+00	0.257	Moderate
%GC	1,740.700	0.00e+00	0.212	Moderate
%GC3	2,658.519	0.00e+00	0.324	Large
*Nc*	2,056.072	0.00e+00	0.251	Moderate
%GC1	181.178	4.55e-40	0.022	Small
%GC2	175.248	8.82e-39	0.021	Small
*nt*	8.119	1.73e-02	0.001	Negligible

*n* = 8,201 for all the genes analyzed.

*H*, Kruskal–Wallis test statistic.

**Figure 1:**
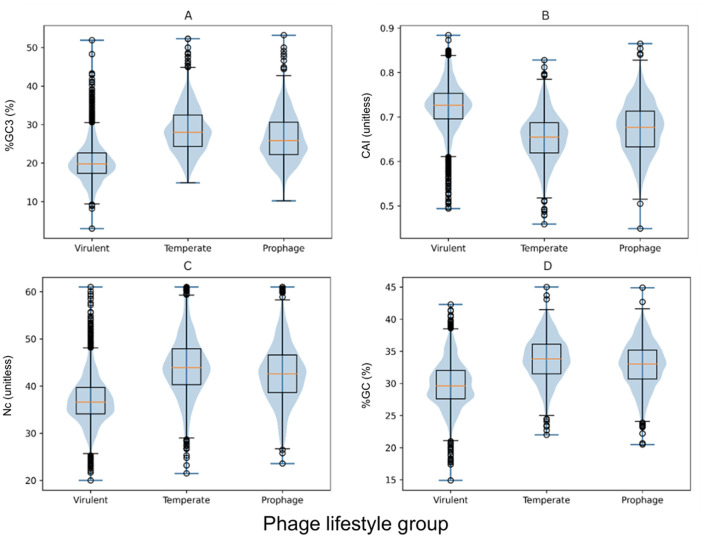
*S. aureus* Phage Lifestyle-associated differences in gene-level codon usage and GC composition. The violin plots represent kernel density estimates of the distributions, illustrating the shape and spread of the data. The embedded boxplots indicate the median (horizontal line) and interquartile range (box), with whiskers extending to the most extreme observations within 1.5 × IQR. **A**–**D** – %GC3, CAI, *Nc* and %GC distributions across the phage lifestyle groups, respectively.

To identify the specific relationships underlying the global differences, pairwise comparisons were conducted for the codon usage metrics with significant Kruskal–Wallis results. These analyses revealed a consistent pattern distinguishing the virulent phages from the temperate and prophage groups (Table 5), with %GC3 showing the most pronounced contrasts: both the temperate and prophage genes exhibited substantially higher %GC3 than those of the virulent phage group (Cliff's δ = −0.728 and −0.584, respectively). A smaller but significant difference was also observed between the temperate and prophage groups, with the former carrying the genes with slightly higher %GC3 (δ = 0.202). The analysis of the CAI-values displayed the opposite trend: the genes of the virulent phage group had markedly higher CAI estimates than those of the temperate phage and prophage groups (δ = 0.677 and 0.484, respectively). A modest increase in CAI was also observed for the genes of the prophage group relative to this estimate for the genes of the temperate phage group (δ = −0.212). The *Nc* patterns mirrored those for GC content: the genes of the temperate phage and prophage groups exhibited higher *Nc*-values than the genes of the virulent phage group (δ = −0.631 and −0.527, respectively). The overall GC content likewise differed substantially, with the genes of the temperate phages and prophages showing higher GC levels than those of the virulent phages (δ = −0.590 and −0.474), while differences between the temperate phage and prophage groups were comparatively minor (δ = 0.143). The first- and second-position GC metrics showed smaller but consistent patterns: the genes of the temperate phage and prophage groups generally had slightly higher %GC1 and %GC2 than those of the virulent phage group, although these differences were modest in magnitude. These lifestyle-associated differences are illustrated in Figure 1, which depicts the distributions of representative gene-level metrics (%GC, %GC3, CAI, and *Nc*) across the three phage lifestyles, consistent with the statistical contrasts reported in Tables 4, 5.

**Table 5:** Pairwise lifestyle contrasts in codon usage and nucleotide composition (Holm-adjusted *p*-values and Cliff's δ effect sizes) among the *S. aureus* virulent and temperate phages, and prophages.

Metric	Group 1	Group 2	*p* (Holm)	Cliff's δ	Effect	Direction
%GC3	Virulent	Temperate	0.00e+00	−0.728	Large	Temperate > Virulent
%GC3	Virulent	Prophage	0.00e+00	−0.584	Large	Prophage > Virulent
CAI	Virulent	Temperate	0.00e+00	0.677	Large	Virulent > Temperate
*Nc*	Virulent	Temperate	0.00e+00	−0.631	Large	Temperate > Virulent
%GC	Virulent	Temperate	9.27e-294	−0.590	Large	Temperate > Virulent
*Nc*	Virulent	Prophage	2.27e-273	−0.527	Large	Prophage > Virulent
CAI	Virulent	Prophage	1.13e-230	0.484	Large	Virulent > Prophage
%GC	Virulent	Prophage	4.49e-221	−0.474	Moderate	Prophage > Virulent
CAI	Temperate	Prophage	4.78e-34	−0.212	Small	Prophage > Temperate
%GC1	Virulent	Temperate	1.70e-33	−0.196	Small	Temperate > Virulent
%GC3	Temperate	Prophage	7.12e-31	0.202	Small	Temperate > Prophage
%GC2	Virulent	Prophage	6.32e-30	−0.171	Small	Prophage > Virulent
%GC2	Virulent	Temperate	5.56e-25	−0.167	Small	Temperate > Virulent
%GC1	Virulent	Prophage	9.53e-23	−0.148	Small	Prophage > Virulent
%GC	Temperate	Prophage	2.43e-16	0.143	Negligible	Temperate > Prophage

### 3.4 Pairwise multivariate differences among phage lifestyles

The Pairwise PERMANOVA analyses confirmed significant differences in the gene-level compositional profiles between all phage lifestyle groups (Supplementary Table S5). The strongest separation was observed between the genes of the virulent and temperate phages (pseudo-*F* = 818.31, *R*^2^ = 0.173, *p* = 0.001), followed by the genes of the virulent phages versus those of the prophages (pseudo-*F* = 476.36, *R*^2^ = 0.109; p = 0.001). In contrast, the difference between the genes of the temperate phages and prophages was much smaller (pseudo-*F* = 53.92, *R*^2^ = 0.014), but remained statistically significant (*p* = 0.001). Multivariate dispersion differed significantly among lifestyles (PERMDISP: *F* = 13.8, *p* < 0.001), indicating that differences in within-group variability may contribute to the observed PERMANOVA signal.

Because PERMANOVA tests differences in multivariate centroids, and is sensitive to differences in dispersion, these results likely reflect both compositional shifts and variability differences among lifestyle groups, and thus, should therefore be interpreted with caution (Anderson, 2001).

### 3.5 Spearman and partial correlation analysis of gene-level codon usage parameters across phage lifestyles

We performed Spearman correlation analyses to characterize the correlation structure among the gene-level length and codon usage metrics across the genomes of the virulent and temperate phages, and those of the prophages. Third codon-position GC content (%GC3) emerged as the dominant determinant of codon usage variation (Supplementary Table S6): specifically, CAI exhibited a very strong negative correlation with %GC3 across all phage lifestyles, including the virulent phages (ρ = −0.79), temperate phage (ρ = −0.76), and prophage genomes (ρ = −0.73) (all FDR-adjusted *p* < 0.001). The narrow confidence intervals indicate highly stable and consistent estimates across datasets. These results demonstrate that variation in CAI is overwhelmingly structured by compositional bias at synonymous third codon positions. However, despite this dominant compositional influence, lifestyle-associated differences in CAI and *Nc* remain consistent across functional gene categories and persist after controlling for GC content, indicating that compositional bias alone does not fully account for the observed patterns.

Similarly, *Nc* displayed consistent moderate-to-strong positive correlations with %GC3 (ρ = 0.44–0.50; all FDR-adjusted *p* < 0.001), further reinforcing the central role of GC3 in shaping codon bias. The CAI–*Nc* relationship was also strong and negative across all lifestyles (ρ = −0.49–−0.56; all FDR-adjusted *p* < 0.001), indicating a conserved structural coupling between codon adaptation and codon bias metrics. In contrast to the dominant influence of GC3, gene length showed comparatively weak associations with codon usage parameters. Log-transformed gene length correlated moderately with %GC2 (ρ = 0.31–0.38; all FDR-adjusted *p* < 0.001). However, correlations between gene length and CAI were weak (ρ = −0.04–−0.09), despite statistical significance in some cases, reflecting large sample sizes rather than strong biological effects. Associations between gene length and *Nc* were similarly weak (ρ = 0.07–0.11).

To determine whether the observed length–codon associations were driven by compositional bias, partial Spearman correlations were computed while controlling for both %GC3 and overall %GC (Supplementary Table S7). Following compositional adjustment, the previously observed correlations between gene length and CAI were no longer significant across all lifestyles (partial ρ = −0.026–−0.008; all FDR-adjusted *p* > 0.10), indicating that the weak raw associations between length and CAI were largely attributable to the underlying GC structure rather than independent length-dependent selection. In contrast, small but statistically significant positive associations between gene length and *Nc* persisted after GC control (partial ρ = 0.039–0.062; FDR-adjusted *p* ≤ 0.048), suggesting a modest independent contribution of gene length to codon bias variation. Notably, the CAI-*Nc* relationship remained moderate and highly significant after controlling for %GC3 and overall %GC (partial ρ = −0.365–−0.417; all FDR-adjusted *p* < 0.001), showing that the structural coupling between CAI and *Nc* cannot be explained solely by GC composition and likely reflects intrinsic properties of codon bias metrics.

In addition, nucleotide composition and codon usage metrics (CAI, %GC, %GC1, %GC2, %GC3, and Nc) were computed genome-wide for the genes of the *S. aureus* phage SA1 (Supplementary Table S8). Overall, The codon usage analysis indicated moderate variability in nucleotide composition and codon bias across the phage genome. Gene lengths were highly variable (mean 937.5 *nt*; range 99 *nt*−11,034 *nt*), reflecting a heterogeneous distribution of coding sequence sizes. The CAI exhibited a relatively narrow distribution (mean 0.752 ± 0.044). The overall GC content was low (mean 26.5%), with a clear positional bias across codon sites (%GC1: 37.5%, %GC2: 24.4%, %GC3: 17.6%), demonstrating a progressive decline from the first to the third codon position. The *Nc* averaged 35.3, and the relatively narrow interquartile ranges, observed across all metrics, indicated a generally uniform codon usage pattern with limited dispersion, despite the presence of some outliers.

### 3.5 Phage lifestyle-associated divergence in gene architecture and synonymous codon usage across functional gene categories

**Figure 2:**
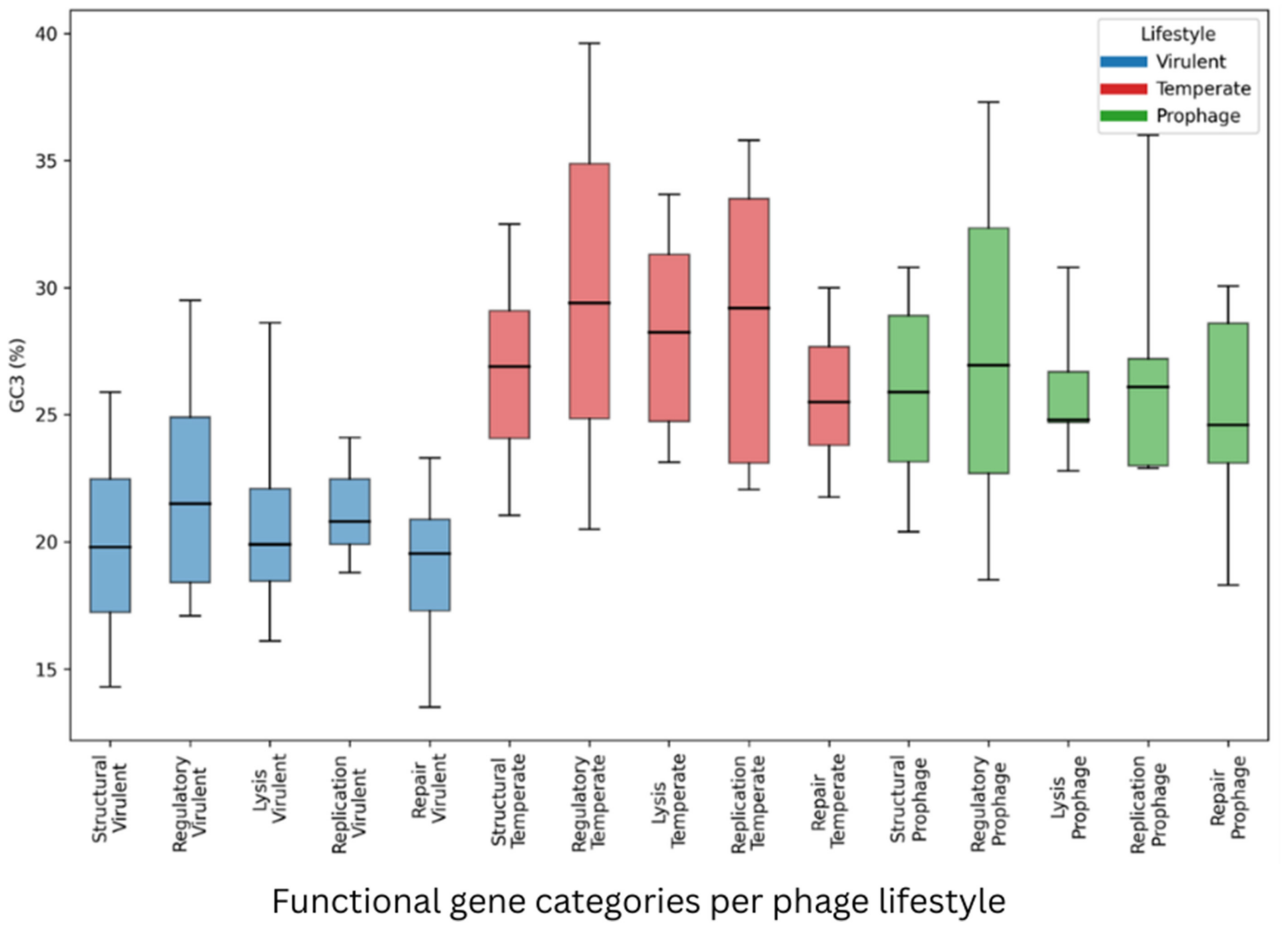
GC3 distributions across the functional gene categories per phage lifestyle. Horizontal line inside box - Median (P50); Bottom of box - Q1 (P25); Top of box - Q3 (P75); Box height - IQR; Lower whisker - P10; Upper whisker - P90

**Figure 3:**
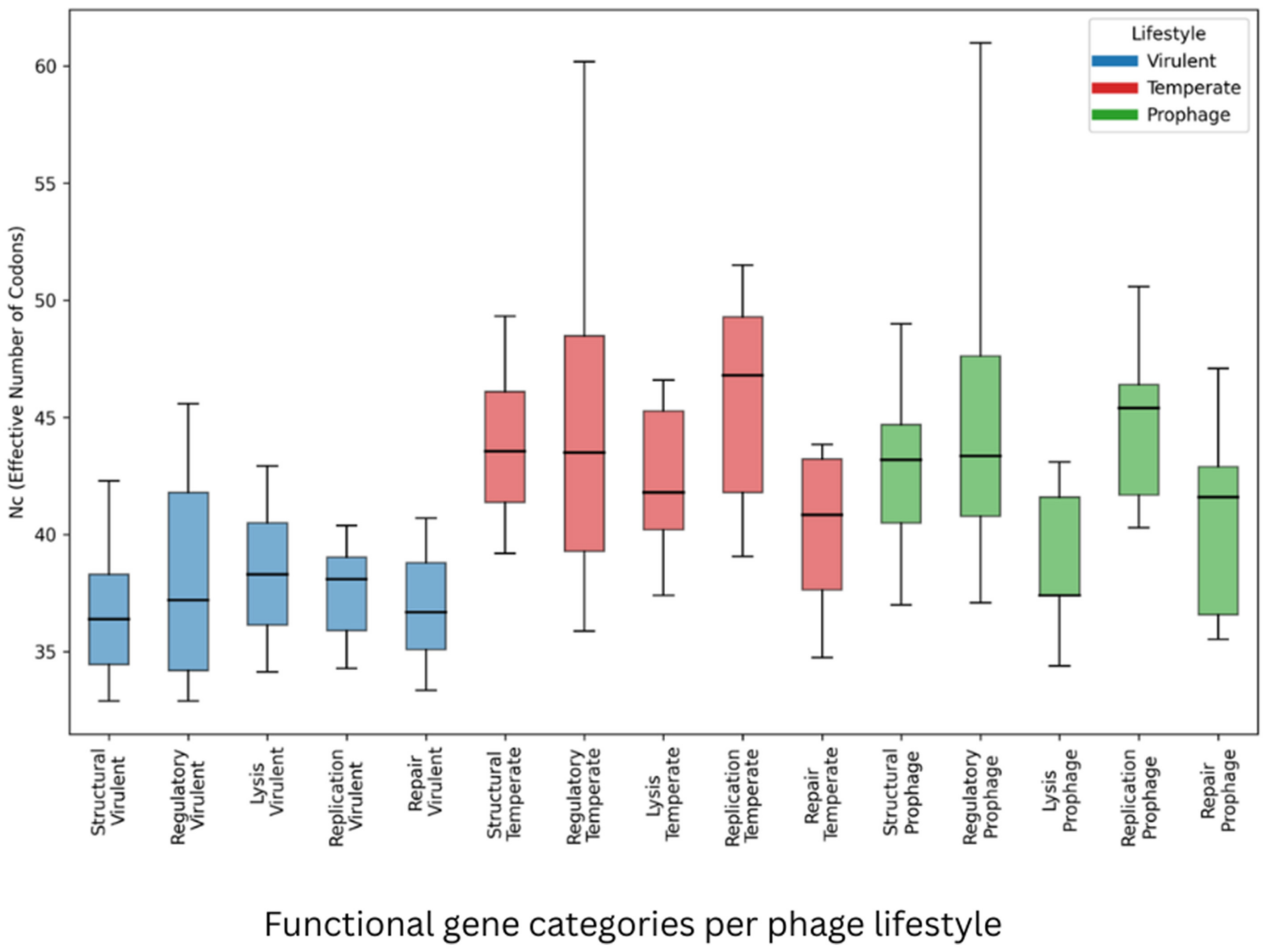
*Nc* distributions across the functional gene categories per phage lifestyle. Horizontal line inside box - Median (P50); Bottom of box - Q1 (P25); Top of box - Q3 (P75); Box height - IQR; Lower whisker - P10; Upper whisker - P90.

**Figure 4:**
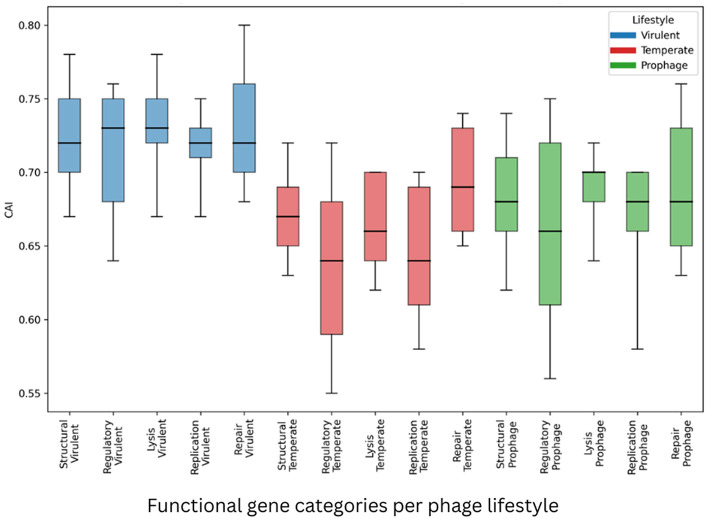
CAI distributions across the functional gene categories per phage lifestyle. Horizontal line inside box - Median (P50); Bottom of box - Q1 (P25); Top of box - Q3 (P75); Box height - IQR; Lower whisker - P10; Upper whisker - P90.

We evaluated whether *S. aureus* phage lifestyle has been associated with systematic differences in gene architecture and synonymous composition. To this end, full distributional properties (percentiles and dispersion) were analyzed across multiple functional gene categories and compositional metrics (Supplementary Tables S9–S15; Figures 2–4), ec-category patterns rather than isolated mean differences. Specifically, the structural, regulatory, lysis, replication, and repair genes, shared across all the phage lifestyle groups, were examined.

Gene length was found to be largely linked to gene functional category; however, within individual categories the percentile structure (IQR and upper percentiles) differed systematically among phage lifestyles (Supplementary Table S9). The structural gene lengths showed broadly overlapping distributions between the virulent and temperate phages (median 879 *nt* vs. 942 *nt*; IQR 1,092 *nt* vs. 1,011 *nt*, respectively). In contrast, the prophage structural genes exhibited a pronounced upper-tail expansion (maximum 8,274 *nt*), exceeding the maxima observed in the virulent and temperate phages groups (6,225 *nt*), indicating occasional enlargement of structural modules. Regulatory gene lengths exhibited the strongest lifestyle-associated divergence. Although median lengths were moderately similar across lifestyles (438 *nt* in virulent, 331.5 *nt* in temperate, and 423 *nt* in prophage genomes), the virulent phages displayed a markedly extended upper tail (P90 = 1,944 *nt*; maximum 3,459 *nt*), substantially exceeding that of the temperate (P90 = 792 *nt*; maximum 2,448 *nt*) and prophage datasets (P90 = 1,038 *nt*). These data reveal a virulent-specific expansion of the regulatory gene length upper tail, indicating the presence of a subset of regulatory proteins whose lengths extend beyond the percentile range observed in the temperate and prophage genomes.

To gain further insight into the specific functions of the outlier regulatory genes with a maximum length of 3,459 *nt*, we analyzed the amino acid sequences of the proteins they encode, using InterPro v108.0 (InterProScan v5.77) and SWISS-MODEL. Based on structural homology, these proteins (YP_009782143.1, YP_009782388.1, YP_009782881.1, and UYL89262.1) were associated with a baseplate wedge component, functioning as a conserved structural element that mediates conformational signal transduction required for tail sheath contraction during infection.

The lysis genes exhibited pronounced dispersion differences, with the temperate phages showing exceptionally broad variability (IQR = 1,143 *nt*; P75 = 1,446 *nt*), whereas the prophage lysis genes were highly constrained (IQR = 183 *nt*; median 303 *nt*). The virulent phage lysis genes were intermediate in variability (IQR = 348 *nt*; median 516 *nt*). The replication genes further demonstrated lifestyle stratification: specifically, the temperate phage replication genes were substantially shorter (median 780 *nt*; IQR = 471 *nt*) than those of the virulent phages (median 1,282.5 *nt*; IQR = 888 *nt*) and prophages (median 1,380 *nt*; IQR = 711 *nt*). The repair genes showed weaker separation, with overlapping medians (712.5 *nt* for virulent; 552 *nt* for temperate; 921 *nt* for prophage) and moderate dispersion differences in these phage lifestyle groups.

Across most functional gene categories, the temperate phages exhibited moderately higher overall GC content than the virulent phages, with the prophages generally occupying intermediate positions (Supplementary Table S10): in the structural genes of the temperate phages, median GC was 35.00% compared to 32.30% determined for those of the virulent phages, while this estimate for the prophages was 34.50%. A similar overall GC pattern was observed across the regulatory genes (35.20% in temperate; 31.50% in virulent; 34.40% in prophage) and the lysis genes (37.00% in temperate; 35.10% in virulent; 32.80% in prophage). The largest difference was revealed in the replication genes, where these genetic loci of the temperate phages exhibited a median GC of 34.20% compared to 30.40% determined for those of the virulent phages, with the respective prophage genes illuminating again intermediate pattern (33.70%). The Repair genes showed weaker separation (30.55% in temperate; 29.50% in virulent; 32.10% in prophage) and overlapping interquartile ranges.

The Codon position-specific GC analysis demonstrated that lifestyle-associated compositional divergence is confined to the third codon position. In contrast to %GC3, both %GC1 and %GC2 showed extensive overlap across lifestyles in all the functional gene categories. For example, in the structural genes, GC1 medians were 44.30% (virulent), 45.30% (temperate), and 44.70% (prophage) (Supplementary Table S11), while GC2 medians were 33.10%, 31.90%, and 32.00%, respectively, with overlapping interquartile ranges (Supplementary Table S12). Similar overlap was observed in the regulatory, lysis, replication, and repair genes, indicating no systematic lifestyle-associated separation at amino acid-constrained positions. In contrast, %GC3 displayed consistent and universal stratification (Supplementary Table S13; Figure 2). Across all five functional categories, the virulent phages exhibited the lowest GC3 medians, the temperate phages—the highest, and the prophages—intermediate values. For the structural, regulatory, and replication genes, GC3 medians were 19.80%/21.50%/20.80% (virulent), 26.90%/29.40%/29.20% (temperate), and 25.90%/26.95%/26.10% (prophage), respectively. In several categories, “virulent GC3” medians approached or fell below “temperate P25” values, indicating limited interquartile overlap and clear distributional displacement. The regulatory genes of the temperate phages exhibited broad dispersion (IQR = 10.03), whereas the replication genes of the virulent phage group were tightly clustered (IQR = 2.58).

The *Nc*-values mirrored GC3 divergence (Supplementary Table S14; Figure 3). In every functional category, the virulent phages exhibited lower *N*c medians (stronger codon bias) than the temperate phage and prophage groups. For the structural, regulatory, and replication genes, *Nc* medians were 36.40/37.20/38.10 (virulent), 43.55/43.50/46.80 (temperate), and 43.20/43.35/45.40 (prophage), respectively. The replication genes showed the largest separation (~8 *Nc* units between “virulent and temperate medians”). The Dispersion patterns paralleled GC3: the regulatory genes of the temperate phage group were highly variable (IQR = 9.18), whereas the replication genes of the virulent phage group were tightly distributed (IQR = 3.13).

CAI distributions further reinforced lifestyle separation (Supplementary Table S15; Figure 4). Across all the functional gene categories, the virulent phages consistently exhibited higher CAI medians than the temperate phage and prophage groups. For the structural, regulatory, and replication genes, CAI medians were 0.72/0.73/0.72 (virulent), 0.67/0.64/0.64 (temperate), and 0.68/0.66/0.68 (prophage), respectively. The replication genes of the virulent phage group displayed extremely narrow dispersion (IQR = 0.02), whereas the regulatory genes of the temperate phages showed broader variability (IQR = 0.09).

### 3.6 Functional gene group differences in *nt* and codon-usage patterns within and among phage lifestyles

We examined whether and/or how the structural, regulatory, lysis, replication, and repair genes differed in the *nt* and codon-usage patterns within and among phage lifestyles. The detailed results obtained from these analyses, exhibiting the observed differences within different phage lifestyle groups, are provided in Supplementary Table S16. It is shown that the *nt* patterns exhibited strong functional differentiation (δ range: −0.738–0.777; Holm-*p* range: effectively 0–0.0073): the largest pooled difference was Structural genes > Regulatory genes (δ = 0.777, Holm-*p* effectively 0), with substantial opposing contrast Lysis genes < Structural genes (δ = −0.738, Holm-*p* = 2.0 × 10^−15^), and Replication genes > Regulatory genes (δ = 0.674, Holm-*p* = 1.07 × 10^−14^).

The CAI patterns differed among functional categories with the small-to-moderate pooled dominance effects (δ range: −0.103–0.296; Holm-*p* range: 3.1 × 10^−6^-0.0135): the strongest CAI pattern separation was Structural genes > Regulatory genes (δ = 0.296, Holm-*p* = 0.0135), followed by Lysis genes > Regulatory genes (δ = 0.278, Holm-*p* = 8.0 × 10^−4^) and Lysis genes > Replication genes (δ = 0.156, Holm-*p* = 3.0 × 10^−6^). The *Nc*-values also showed significant functional-group differences in pooled analyses (δ range: −0.321–0.289; Holm-*p* range: 1.81 × 10^−7^ to 0.0234): the largest pooled effect was Lysis genes < Regulatory genes (δ = −0.321, Holm-*p* = 0.0222).

The overall GC content exhibited the large-pooled effects (δ up to 0.728; Holm-*p* down to 2.22 × 10^−15^) across the functional gene categories within the phage lifestyle groups. The strongest contrasts consistently involved the repair gene group, with Lysis genes > Repair genes (δ = 0.728, Holm-*p* = 2.22 × 10^−15^), Regulatory genes > Repair genes (δ = 0.653, Holm-*p* = 2.62 × 10^−10^), and Structural genes > Repair genes (δ = 0.632, Holm-*p* = 4.42 × 10^−11^). These GC contrasts commonly remained significant within all phage lifestyles. GC1 differences were similarly driven by contrasts against the repair genes, with Regulatory genes > Repair genes (δ = 0.592, Holm-*p* = 9.87 × 10^−10^), and Structural genes > Repair genes (δ = 0.528, Holm-*p* = 1.35 × 10^−8^). The Lysis genes > Repair genes contrast also remained significant (δ = 0.420, Holm-*p* = 7.0 × 10^−4^). The GC2 patterns displayed the strongest separations observed in the dataset (very large pooled effects; δ up to 0.915; Holm-*p* effectively 0 for multiple contrasts): the top effects were Lysis genes > Repair genes (δ = 0.915), Lysis genes > Replication genes (δ = 0.890), and Lysis genes > Regulatory genes (δ = 0.866), all with extremely small Holm-adjusted *p*-values (reported as 0 due to numerical underflow). The GC3 estimates showed significant but comparatively smaller pooled effects (|δ| up to 0.321; Holm-*p* range: 6.39 × 10^−5^-0.0056). The largest contrast was Structural genes < Regulatory genes (δ = −0.321, Holm-*p* = 0.0024), followed by Regulatory genes > Repair genes (δ = 0.299, Holm-*p* = 0.0056).

To determine whether the shared functional gene categories differed in the *nt* and codon usage patterns between phage lifestyles, pairwise comparisons were conducted among the phage lifestyle groups. Here, only contrasts with Holm-adjusted *p* < 0.05 are reported (Supplementary Table 17). Effect sizes are expressed as Cliff's δ, where |δ| ≥ 0.474 indicates a large effect, and |δ| ≥ 0.80 indicates a very large dominance effect. The lysis genes showed pronounced lifestyle effects: these genes of the virulent phages had significantly higher CAI than those of the temperate phages (δ = 0.80, *p* = 3.03 × 10^−7^) and the prophages (δ = 0.748, *p* = 1.26 × 10^−6^). Conversely, %GC3 was markedly lower in the above genes of the virulent phages relative to both those of the temperate phages (δ = −0.794) and the prophages (δ = −0.73). For the lysis genes, *Nc* was significantly lower across the virulent phage group compared to the temperate phage group (δ = −0.564), consistent with stronger codon bias. The *nt*-values were greater for these genes of the virulent and temperate phages relative to those of the prophages (δ = 0.713 and 0.627, respectively).

The regulatory genes illuminated some of the strongest lifestyle differentiation. These genes of the virulent phages showed dramatically higher CAI than those of the temperate phages (δ = 0.873) and prophages (δ = 0.823). However, the overall %GC and %GC3 were substantially lower in the above genes of the virulent phages compared to those of both other lifestyles (|δ| up to 0.862). The *Nc*-values were also markedly reduced for the regulatory genes across virulent phage lifestyle (δ ≈ −0.82), indicating pronounced codon bias.

The repair genes displayed intermediate-to-large lifestyle effects, with those of the virulent phages having higher CAI than both the temperate phages and prophages (δ = 0.474–0.692). These genes of the virulent phages showed markedly lower GC3 relative to their counterparts from the temperate phage (δ = −0.904) and prophage (δ = −0.942) lifestyles. *Nc* was also significantly lower in the repair genes of the virulent phages (δ down to −0.828). The differences were also observed in the *nt* patterns between the temperate phages and prophages (δ = −0.496), with the prophage repair genes being longer.

The replication genes showed the most extreme lifestyle-associated divergence: these genes from the virulent phage group had exceptionally higher CAI than those from the groups of the temperate phages (δ = 0.934, *p* = 4.54 × 10^−8^) and prophages (δ = 0.947). Conversely, the overall %GC, %GC2, and %GC3 were significantly lower in in the above genes of the virulent phages (|δ| up to 0.908). The *Nc*-values exhibited extremely large effects (δ ≈ −0.94 vs both lifestyles), indicating very strong codon bias, while the *nt*-values, for these genes, were greater in the virulent phages compared to the temperate phages (δ = 0.762).

The structural genes mirrored the patterns of the replication genes: these loci from the virulent phage group exhibited markedly higher CAI than those from the temperate (δ = 0.922) and prophage (δ = 0.862) groups. At the same time, the overall %GC and %GC3 were substantially lower in these genes of the virulent phages (GC3 δ up to −0.932). *Nc* differences were among the strongest observed in the dataset (δ = −0.95 vs Temperate; δ = −0.936 vs Prophage), indicating pronounced codon bias in the structural genes of the virulent phages.

### 3.7 Codon usage patterns of functional genes in *S. aureus* virulent jumbo phage SA1

The analysis of genome annotation patterns in the *S. aureus* jumbo virulent phage SA1 revealed that a high proportion of genes encoded hypothetical proteins (77.9%), alongside an apparent absence of annotated regulatory genes. This observation likely reflects limitations of current annotation frameworks rather than a true lack of regulatory functions. Notably, phage regulatory proteins are often highly divergent, poorly conserved, or encoded by small and multifunctional open reading frames that may escape detection by homology-based approaches, particularly given the limited representation of jumbo phage genomes in the current DNA sequence databases. The *nt*-pattern analysis of the functional gene groups of the jumbo phage SA1 revealed notable differences in gene size distribution (Supplementary Table S18). In particular, the structural genes were the longest on average (mean: 2210.4 *nt*) and exhibited the greatest variability (IQR: 1,503 *nt*), reflecting a broad range of gene lengths. The replication genes showed intermediate lengths (mean: 1,834 nt) with relatively low dispersion (IQR: 464.25 nt), while the repair genes were shorter on average (mean: 1,089.75 *nt*), but remained moderately variable (IQR: 872.25 *nt*). In contrast, the single lysis gene encoding endolysin (*n* = 1) displayed a fixed length of 837 *nt*, resulting in no observed variability.

The GC content analysis demonstrated clear differentiation (Supplementary Table S19), with the endolysin gene displaying the highest GC content (36.2%), which markedly exceeded that of the other functional groups (means: 27.6%−28.9%). The structural genes showed the greatest variability in overall GC% (IQR = 3.45), while the replication and repair genes were more constrained (IQR ≤ 1.53). The similar GC trends were observed across codon positions: at the first codon position (GC1), the lysis gene again showed the highest value (41.6%), while the repair genes exhibited the greatest dispersion (mean = 38.28%; IQR = 8.08) (Supplementary Table S20); at the second position (GC2), the endolysin gene appeared to be distinctly enriched (47.0%) compared to the other groups (means: 26.9%−31.0%), whereas the structural and repair genes showed broader variability (means: 30.99% and 29.18%; IQR: 6.85 and 5.62, respectively) (Supplementary Table S2). The third codon position (GC3) remained comparatively low across all the gene categories (means: 16.4%−17.2%), with the lysis gene exhibiting 20.1%, while the structural genes (mean: 16.42%) displayed the widest spread (IQR = 4.05) (Supplementary Table S22). Overall, the CAI estimates were relatively high across all the functional gene groups of this jumbo phage (means: 0.73–0.76). The structural, replication, and repair genes exhibited the comparable CAI distributions (means ≈0.76), with moderate variability (IQR: 0.02–0.04), whereas CAI did not exceed 0.73 for the lysis gene (Supplementary Table S23). In addition, the lysis gene exhibited the lowest *Nc* value (32.4), indicative of stronger codon usage bias, whereas the replication genes had the highest mean *Nc* (35.24). The structural and repair genes showed intermediate *Nc* values with modest variability (Supplementary Table S24).

## 4 Discussion

### 4.1 *S. aureus* phage lifestyle-associated genomic heterogeneity reflects distinct evolutionary regimes

In this study, we demonstrate that lifestyle is a major determinant of genomic heterogeneity, architecture, and codon usage structure in *S. aureus* phages. By integrating the whole-genome similarity metrics, genome size distributions, gene-level compositional analyses, multivariate comparisons, and functional stratification, it is shown that the populations of the virulent and temperate phages of this host species, and its prophages occupy distinct evolutionary regimes. The within-group genome comparisons revealed marked differences in how heterogeneity is structured across phage lifestyles: among the genome pairs with detectable similarity, the temperate phages exhibited the greatest divergence (mean CGD = 0.793), followed by the prophages (0.735), whereas the virulent phages showed comparatively lower divergence within the aligned regions (0.586). However, when complete divergence events were included in the analysis, the virulent phages displayed the highest overall heterogeneity due to a large proportion of the NSS pairs (59.1%). This distinction is conceptually consistent with the previous observations. In particular, the studies have shown the pronounced within-cluster/pangenome relationships in staphylococcal lytic phages driven by extensive recombination and modular exchange, contrasted with between-cluster comparisons often exhibiting limited detectable (Oliveira et al., 2019) versus high (Cui et al., 2017) similarities at the nucleotide level. Besides, it was demonstrated that *S. aureus* lytic phages with myovirus-like morphology can segregate by signature distance, including cases in which phages of comparable genome size exhibit no significant nucleotide homology (Deschavanne et al., 2010). This finding parallels the elevated proportion of NSS pairs observed in the present study, also showing that, unlike the virulent phages, exhibiting the greatest within-group divergence, the temperate phages demonstrated no NSS events, while the prophages—only the rare ones (2.1%). This pattern appears consistent with the temperate-enriched cluster previously described, which exhibits high in-group diversity yet remains genomically connected through gene-content similarity within a mosaic and subclustered architecture (Oliveira et al., 2019).

The genome size distributions were strongly lifestyle-structured (ε^2^ ≈ 0.22): the genome size variations of the *S. aureus* virulent phages exhibited extreme heterogeneity and clear bimodality (~17 kb−18 kb vs. ~130 kb−140 kb clusters). This suggests divergent evolutionary strategies within obligately lytic phage lineages: compact genomes potentially optimized for rapid replication cycles and larger, complex genomes encoding expanded structural and replication machinery. In contrast, the temperate phages illuminated highly constrained genome sizes (median ~43 kb; IQR 1.2 kb). The *S. aureus* phage “lifestyle-structured” tendencies, observed in our study, provide additional strong evidence for the genome size concentration of the temperate phages within ~40 kb−50 kb range, whereas that of the obligately lytic phages of this host species span multiple distinct genome-size lineages (Ingmer et al., 2019). In the temperate phages, such a genome-size compactness likely reflects selective optimization for lysogeny-compatible organization, where essential modules for integration, maintenance, and regulated induction must be preserved without excessive genomic burden. The genomes of the *S. aureus* prophages appear to be significantly larger and more variable in size (median ~60 kb; IQR ~13 kb). Their size distribution is broadly consistent with the previously reported prophage genome ranges (13 kb−81 kb) (Ene et al., 2021; Naorem et al., 2021; Liang et al., 2025). Notably however, we identified an unusually large prophage (>130.5 kb; coordinates: 2,196,978–2,327,572) within the genome of the *S. aureus* strain from China (NZ_CP018629.1). This finding closely parallels a previously described ~127-kb prophage-like element in *S. aureus*, showing similarity to a *Staphylococcus epidermidis* prophage, and interpreted as the result of cross-species acquisition followed by dissemination among some *S. aureus* lineages (Ingmer et al., 2019). Thus, lifestyle influences not only the magnitude of genomic diversity but also its topology, with the temperate phages of this species diversifying within a connected genomic space, whereas their virulent counterparts can include discontinuous, disconnected genomic clusters.

Furthermore, the correspondence between genome size bimodality and family-level taxonomy in the *S. aureus* virulent phages suggests that genome size distribution may be partially shaped by underlying taxonomic structure. Accordingly, some apparent lifestyle-associated patterns could be influenced by evolutionary differences among distinct virulent phage lineages. The observed genome size-based clustering of phages into the *Rountreeviridae* and *Herelleviridae* highlights the need for further investigation to disentangle taxonomic effects from functional adaptation.

In addition, the positive association between genome size and tRNA content in the larger virulent phages suggests an increased capacity for encoding auxiliary translational components. The restriction of tRNA carriage to the larger genome size class further indicates that this trait is lineage-specific rather than a general feature of these *S. aureus* virulent phages. This pattern is consistent with previous studies showing that phage-encoded tRNAs are often associated with genome expansion and may help compensate for differences in codon usage between phages and their hosts (Bailly-Bechet et al., 2007). However, while other scenarios may exist, the lack of association between tRNA presence and CAI in our dataset suggests that tRNA acquisition does not directly mirror codon adaptation as captured by standard metrics. Instead, it may reflect broader functional or evolutionary roles, such as supporting translation under host-imposed constraints, contributing to infection efficiency, or mediating interactions with host regulatory systems, as proposed in previous studies (Delesalle et al., 2016; van den Berg and Brouns, 2025). Nevertheless, potential inconsistencies in phage genome annotations within the GenBank database should be considered. In particular, it should be acknowledged that despite restricting the dataset to the well-annotated *S. aureus* phage genomes, residual annotation errors may persist and could affect the inferred distribution of tRNA genes.

The *S. aureus* temperate phages appear to form a recombinational continuum, mosaic and diverse, yet retaining sufficient shared modules (e.g., integrases and repressors) to preserve detectable homology within a connected similarity network. In contrast, the virulent phages include deeply divergent lineages lacking detectable similarity under uniform alignment criteria, consistent with episodic diversification, host switching, and/or acquisition of distinct genomic architectures. The *S. aureus* prophages occupy an intermediate position, consistent with derivation from their “temperate ancestors” followed by host-mediated homogenization or stabilization after chromosomal integration. Furthermore, it needs to be considered that the intact prophages may include elements undergoing early mutational decay, as well as others potentially transitioning toward host-associated functional integration, which together could explain their intermediate compositional metrics. In addition, it can be thought that lifestyle influences not only the magnitude of genomic diversity, but also its topology, with the temperate phages diversifying within a connected genomic space, whereas the virulent phages may comprise discontinuous and genomically disconnected clusters.

Collectively, these findings reveal a fundamental lifestyle-associated dichotomy in the evolutionary regimes of the *S. aureus* phages: temperate phages diversify within a recombinational continuum, remaining genomically connected despite high internal divergence, whereas virulent phages comprise deeply divergent lineages that often lack detectable similarity and form discontinuous genomic clusters. Prophages occupy an intermediate position, consistent with their “temperate ancestry” followed by host-mediated stabilization after chromosomal integration. Thus, we show that *S. aureus* phage lifestyle shapes not only the magnitude of genomic diversity, but also its topological organization within genome similarity space, distinguishing connected diversification from genomic discontinuity under contrasting evolutionary strategies.

### 4.2 GC3-driven synonymous divergence and multivariate profiles separate phage lifestyles.

Across the full gene set, the dominant lifestyle-associated signal was concentrated at synonymous sites, with the %GC3 pattern explaining a large fraction of ranked variance (ε^2^ ≈ 0.324), while the %GC1 and %GC2 patterns showed only small effects (ε^2^ ≈ 0.02 each). Thus, lifestyle divergence appears to be primarily “synonymous/compositional,” rather than reflecting widespread divergence at amino-acid-constrained positions. It is noteworthy that the same ordering persisted genome-wide and within each functional module examined: the genes of the virulent phages exhibited the lowest %GC3 versus the highest %GC3 of the genes of the temperate phages, while the prophage genes fell into an intermediate category. These patterns argue against a single-category artifact, indicating instead a pervasive compositional axis expressed across the replication, structural, and lysis functions. Such a GC3-focused axis is also consistent with broader comparative phage genomics showing that non-random patterns of GC3 can occur across phage genomes and that GC3 variation may coexist with, yet be partly separable from, signals of host-codon adaptation (Lucks et al., 2008).

Within lifestyles, a strong negative coupling between CAI and %GC3 was observed (ρ ≈ −0.73–−0.79). Although informative, this relationship should be interpreted conservatively in low-GC genomic contexts: in particular, it must be indicated that preferred-codon tables can be intrinsically AT- or GC-skewed at third positions, and the direction of CAI–GC3 association can differ across hosts with contrasting preferred-codon composition (Lucks et al., 2008). In this study, additional support for a translation-associated component comes from the persistence of CAI–*Nc* coupling after GC control (partial ρ ≈ −0.365–−0.417), consistent with the codon-usage structure not being fully reducible to base composition. Mechanistically, such a structure is plausible, because synonymous codon bias can generate measurable fitness advantages in highly expressed genes (Brandis and Hughes, 2016). At the same time, it needs to be emphasized that “translation optimization” is multilayered: for example, phages with relatively inefficient translation initiation may experience weaker selection on elongation-related codon adaptation, and lifestyle (virulent vs temperate) has been implicated as a mediator of selection intensity on translation initiation and elongation (Prabhakaran et al., 2015).

In our study, functional and multivariate analyses further reinforced lifestyle-associated structuring. Stratification in %GC3, CAI, and *Nc* remained robust under functional partitioning, with the replication genes consistently among the strongest discriminators, while the structural and lysis modules also displayed consistent shifts. This module-consistent behavior aligns with the broad phage-scale observations that regions encoding high-copy virion components (e.g., head/tail or capsid/tail proteins) often exhibit pronounced codon bias toward host-preferred codons (Lucks et al., 2008). The strong replication-module separation is also compatible with comparative evidence that the replication-associated features (including the polymerase usage and mutational pressure) can shape synonymous GC and codon-distribution patterns in ways that differ between the temperate and virulent systems (Kunisawa et al., 1998). At the gene-profile level, PERMANOVA supports the same biological picture: separation is strongest between the virulent and temperate phages (*R*^2^ ≈ 0.165), moderate between the virulent and prophage lifestyles (*R*^2^ ≈ 0.113), and minimal between the temperate and prophage lifestyles (*R*^2^ ≈ 0.009). Because PERMANOVA tests differences in multivariate centroids, this interpretation is strengthened by explicitly considering dispersion, particularly in unbalanced settings (Anderson, 2001). The temperate-like placement of the prophage genes, observed in our study, is consistent with the established expectations that integrated prophages experience extensive host-associated processes (mutation, rearrangement, deletion and modular exchange), and that foreign DNA can “ameliorate” toward resident-genome compositional properties over time (Canchaya et al., 2003). More generally, the comparative analyses of phage tRNA carriage and codon usage suggest that virulent phages can display larger compositional gaps to hosts and stronger codon usage bias than temperate phages, potentially compensated by selective recruitment of phage-encoded tRNAs. This is an external line of evidence that fits the directionality observed here while still allowing multiple contributing mechanisms (Bailly-Bechet et al., 2007).

Finally, the direction and magnitude of codon-adaptation signals may not be universal across host systems: a large lifestyle-stratified analysis of *Listeria monocytogenes* phages reported higher CAI for temperate phages, but stronger codon usage bias (lower *Nc*) for virulent phages, with a strong and negative CAI–%GC3 correlation (Kobakhidze et al., 2025). Together with the earlier analyses of staphylococcal phages that identified mutation bias as a major driver, while still detecting contributions from translational selection and gene-level features, our findings underscore that the GC3-centered composition often represents a dominant axis of variation. In contrast, the CAI/*Nc* patterns may reflect partially distinct strategies and host-dependent coupling.

### 4.3 Lifestyle-dependent codon bias in *S. aureus* phages

In our comparative analysis, the genes of the *S. aureus* virulent phages show the strongest signals of potential translational optimization. Specifically, they exhibit higher CAI-values and lower *Nc*-values than those from the temperate phage or prophage genomes, with especially tight codon-usage distributions across the replication, structural, and lysis modules. This pattern matches the expectation that phage genes required in larger amounts—e.g., capsid and DNA replication proteins—should preferentially use host-preferred codons to maximize translation efficiency (Bahir et al., 2009; Sirihongthong and Auewarakul, 2025). Indeed, the previous studies report that bacteriophage genomes often closely match their hosts' codon usage representing an intuitive strategy to exploit the host's translation machinery (Sirihongthong and Auewarakul, 2025); in phages, the highly expressed structural proteins (capsid, tail, etc.) show the greatest codon bias toward the host-preferred codons (Bahir et al., 2009; Prabhakaran et al., 2015). Likewise, the *S. aureus* virulent phages, in our dataset, exhibit stronger codon adaptation than the temperate phages, as reflected by their higher CAI values and overall stronger codon bias. This pattern is consistent with stronger translational selection in virulent phages relative to their temperate counterparts. Although CAI is strongly influenced by GC3, as demonstrated by the robust correlations observed across all lifestyle groups, the persistence of lifestyle-associated differences after controlling for compositional bias suggests that additional selective forces may contribute to codon usage patterns. In particular, the combined pattern of higher CAI, lower *Nc*, and reduced GC3 in virulent phages supports the interpretation of enhanced translational selection, although this inference should be still made with caution, given the substantial contribution of underlying nucleotide composition. Similar trends have been reported in other phage-host systems: for example, staphylococcal virulent phages were shown to possess weaker mRNA secondary structures illuminating higher CAI values than the temperate phages,—the features interpreted as facilitating more efficient translation during the lytic cycle (Prabhakaran et al., 2015). Lifestyle-associated codon-usage differences were also observed in *Listeria* phages (Kobakhidze et al., 2025).

Importantly, our GC-controlled analyses confirm that this signal is not merely a byproduct of base-composition bias. The CAI–*Nc* correlation remains moderately strong (partial ρ ≈ −0.37–−0.42) even after statistically controlling for overall GC and third-position GC content. This implies that lifestyle-associated codon differences go beyond simple GC effects and reflect genuine, structured codon preferences. In other words, the *S. aureus* virulent phages don't just happen to have different GC content—they specifically use a non-random set of codons aligned with host tRNA abundances, as expected under translational selection (Bahir et al., 2009; Sirihongthong and Auewarakul, 2025). Such an optimization of codon usage is well recognized in prokaryotes and their phages: genes with high expression or high copy number tend to converge on host-preferred codons, improving elongation efficiency (Bahir et al., 2009; Sirihongthong and Auewarakul, 2025).

Finally, we note an intriguing finding that the regulatory genes of the *S. aureus* virulent phages (transcription factors, anti-repressors, etc.) include an unusually large tail of very long genes compared to those of the temperate phages and prophages of this host species. Rather than over-interpret this singular trend, we present it as hypothesis-generating: one possibility is that some *S. aureus* virulent phages encode multi-domain regulatory proteins or fusion proteins that combine multiple functions, given that, for example, *Listeria* phages encode dual-domain regulators that control both phage and host responses (Azulay et al., 2022); alternatively, “long” regulators could carry additional domains for host takeover functions or even represent annotation artifacts of fused Open reading frames. Distinguishing among these explanations will require detailed domain-architecture analysis and functional testing of these large regulators. In summary, our findings highlight that the *S. aureus* virulent phages are characterized by stronger codon-usage adaptation to the host, suggesting new directions for exploring unusual features (like regulator length) that may underpin their lytic lifestyle.

Phages with genome sizes between 200 kb and 500 kb are generally classified as jumbo (or giant) phages (Yuan and Gao, 2017). The jumbo phage SA1, included in this study, encodes a multi-subunit RNA polymerase and DNA polymerases, consistent with the partial transcriptional and replicative autonomy described for ϕKZ-related jumbo phages. Nevertheless, the host machinery is still co-opted during infection, supporting a model of semi-independent, host-assisted replication (Zhang et al., 2022). Within this framework, SA1 is best interpreted not as a contradiction to the lifestyle-associated trends identified here, but as a biologically informative extreme within the virulent phage spectrum. Despite its pronounced genomic divergence and large genome size, SA1 remains directionally consistent with the broader virulent group in this dataset, exhibiting the longer coding sequences, lower %GC and %GC3, higher CAI, and stronger codon usage bias (i.e., lower *Nc*) than temperate phages and prophages. This pattern aligns with evidence that phage codon usage is shaped by host-driven translational selection, particularly for highly expressed structural genes, and that lifestyle influences translational optimization in *Staphylococcus* phages (Lucks et al., 2008). However, this interpretation should be treated still with caution. The relationship between lifestyle and codon adaptation is not universal across phage-host systems; for example, virulent phages may display stronger codon bias without consistently higher CAI than temperate counterparts (Kobakhidze et al., 2025). In addition, jumbo phages frequently encode auxiliary tRNAs and transcription-associated functions that can reduce dependence on host machinery, complicating a purely host-driven interpretation of codon usage (Nazir et al., 2021). Finally, mechanistic inferences remain limited because SA1 represents a single genome and, as is typical for jumbo phages, contains a high proportion of hypothetical proteins (Zhang et al., 2022).

Along with these highlights, several limitations of this study should also be acknowledged. The analyses relied on publicly available genome sequences and computationally identified prophages, which may include annotation inconsistencies or incomplete assemblies that could affect gene-level estimates. Furthermore, in our study, a potential limitation of the treating genome pairs with NSS as maximally divergent may overestimate evolutionary distances, particularly where homology falls below BLAST detection thresholds. Besides, homologous relationships may persist at the protein structural level that are not captured by primary nucleotide sequence analyses, especially when BLAST-inferred NSSs are interpreted as evidence of evolutionary divergence. However, as this approach was used primarily to ensure the selection of genetically diverse genomes, it is unlikely to affect the interpretation of codon usage patterns in our study. Although the dataset was balanced across lifestyles, the sample size represents only a subset of the known diversity of *S. aureus* phages. Moreover, the codon-usage analyses based on 1,517,117 codons (4,964 CDSs) from a single host reference (*S. aureus* strain MW2) may not fully capture strain-specific translational preferences. This limitation should be considered, as some phages may be adapted to specific sequence types (STs) of *S. aureus* rather than to species-wide ST diversity. Furthermore, given that codon frequencies were derived from a single-strain genome, the stochastic nature of codon usage in very short genes (< 300 *nt*) may contribute to increased variance in *Nc* and CAI estimates. In contrast, incorporating multi-strain genomic codon frequency datasets may provide improved resolution of the subtle selective pressures acting on these smaller open reading frames. It should also be acknowledged that our gene-level analyses assume independence among the coding sequences despite their nested organization within the phage genomes, which may introduce pseudoreplication and inflate statistical significance. Future studies using hierarchical or mixed-effects modeling approaches could more explicitly account for gene-genome dependency and further refine inference. Also, further investigations incorporating diverse phage families, episomal phages, and additional jumbo phages would meaningfully extend these preliminary findings and provide a more comprehensive understanding of lifestyle-dependent codon usage patterns in *S. aureus* phages.

## 5 Conclusion

Bacteriophage lifestyle is a key determinant of genome architecture and codon usage in *S. aureus* phages. The temperate phages exhibit small, tightly constrained genomes and form a continuous recombinational network without complete genomic discontinuities, whereas the virulent phages show greater genomic heterogeneity, frequent NSS events, and a strongly bimodal genome-size distribution. The bimodal genome size distribution among the *S. aureus* virulent phages aligns with family-level taxonomy, suggesting that genome size variation within these phages is associated with family-level differences. The association of tRNA carriage with larger genome size in the *S. aureus* virulent phages suggests a lineage-specific increase in translational capacity that is not directly linked to codon adaptation but reflects broader functional or evolutionary roles. The prophages of this host species occupy an intermediate position, with the larger and more variable genomes than the temperate phages. The strongest lifestyle-associated signal occurs in the synonymous composition, particularly at third-codon-position GC content. The virulent phage genes display lower %GC3, higher CAI values, and lower *Nc* values, indicating stronger codon bias, and possibly, translational optimization toward the host. The temperate phage genes show the opposite pattern, while the prophage genes remain intermediate but closer to those of the temperate phages. These differences persist across the functional modules and remain significant after controlling for base composition, supporting selection on codon usage beyond mutational bias. Across all phage lifestyles, CAI is strongly negatively correlated with %GC3, suggesting a trade-off between translational efficiency and GC-rich synonymous codon usage. It is also suggested that lifestyle-associated translational optimization can appear to be conserved even in expanded genomes of *S. aureus* virulent phages, and that lifestyle exerts a stronger influence on codon-usage patterns than genome size alone in these organisms. Together, these patterns point to two contrasting evolutionary regimes: modular, connected diversification in the temperate phages and episodic, discontinuous divergence in virulent phages. Enhanced host-codon adaptation in the virulent phages likely facilitates rapid lytic replication, whereas the more GC-rich and compositionally variable genomes of the temperate phages and prophages are consistent with long-term persistence through lysogeny. Codon usage and %GC3 profiles may therefore provide useful indicators of phage lifestyle and host adaptation.

## Data Availability

In this study, the genome DNA sequences for the *S. aureus* strains and phages, were used, which are publicly available in the NCBI GenBank database (https://www.ncbi.nlm.nih.gov/). The corresponding GenBank accession numbers, for the above sequences, are listed in Supplementary Tables S1 and S2, respectively.
