# Supplementary material for: Lifestyle shapes genome architecture and codon usage bias in Staphylococcus aureus bacteriophages, suggesting stronger host adaptation in their virulent lineages
Source: Front Microbiol. 2026 May 20;17:1829197. doi: 10.3389/fmicb.2026.1829197 (PMC13230107; doi:10.3389/fmicb.2026.1829197)
Supplement: Supplementary file 1 [file Data_Sheet_1.pdf]

SUPPLEMENTARY MATERIAL

Supplementary Table S1. The prophages identified across the genomes of diverse *S. aureus* strains, using PHASTER

No.	Host strain genome GenBank acc. code	Host strain designation	Host strain isolation source	Prophage genome coordinates	Prophage genome size (bp)	Systematic affiliation of prophage determined by TaxMyPhage/INPHRARED (GenBank acc. Code)
1	NC_007795.1	NCTC 8325	N/A	1451176-1523261	72086	Staphylococcus phage phi 12 (AF424782)
2	NC_007795.1	NCTC 8325	N/A	1907832-1966929	59098	Staphylococcus phage phi 11 (AF424781)
3	NC_007795.1	NCTC 8325	N/A	2029598-2077786	48189	Staphylococcus phage phi 13 (AF424783)
4	NZ_CP011526.1	DSM 20231	N/A	834262-886092	51831	Staphylococcus phage SH-St 15644 (MG770897)
5	NZ_CP064365.1	PartF-Saureus-RM8376	N/A	144730-211043	66314	Staphylococcus phage phiMR11 (AB370268)

6	NZ_AP025249.1	IDSA1	Pus	1142246-1205168	62923	Staphylococcus phage YMC/09/04/R1988 (KF598856)
7	NZ_AP025249.1	IDSA1	Pus	2156282-2204208	47927	Staphylococcus phage 85 (AY954953)
8	NZ_CP030138.1	M48	N/A	1998516-2061960	63445	Staphylococcus phage SA13 (JX094501)
9	NZ_CP018629.1	MRSA107	N/A	2196978-2327572	130595	Staphylococcus phage phiBU01 (KF831354)
10	NZ_CP013959.1	V605	Blood	1195949-1241843	45895	Staphylococcus phage phi2958PVL (AP009363)
11	NZ_AP019306.1	TUM9463	N/A	696094-752852	56759	Staphylococcus phage SA13 (JX094501)
12	NZ_AP019306.1	TUM9463	N/A	509878-583327	73450	Staphylococcus phage P954 (GQ398772)
13	NZ_CP013957.1	V521	Blood	2034171-2097539	63369	Staphylococcus phage phiNM3 (DQ530361)
14	NZ_CP013957.1	V521	Blood	2137468-2214415	76948	Staphylococcus phage phiETA3 (AP008954)
15	NZ_AP019306.1	TUM9463	N/A	2705519-2771052	65534	Staphylococcus phage P630 (KT809369)
16	NZ_CP012011.1	HC1340	From a patient associated to	1685505-1745682	60178	Staphylococcus phage 13 (AF424783)

			Home Care assistance			
17	NZ_CP012011.1	HC1340	From a patient associated to Home Care assistance	2223079-2274626	51548	Staphylococcus phage 12 (AF424782)
18	NZ_CP009681.1	Gv69	Patient with hospital-associated wound infection	374348-424195	49848	Staphylococcus phage SA97 (KJ716334)
19	NZ_CP009681.1	Gv69	Patient with hospital-associated wound infection	904421-967446	63026	Staphylococcus phage 12 (AF424782)
20	NZ_CP009681.1	Gv69	Patient with hospital-associated wound infection	1390053-1433415	43363	Staphylococcus phage SA345ruMSSAST8 (MH401416)
21	NZ_CP009681.1	Gv69	patient with hospital-associated wound infection	2225287-2274127	48841	Staphylococcus phage SA345ruMSSAST9 (MH401416)
22	NZ_CP015447.2	M92	Nasal swab	2101483-2152901	51419	Staphylococcus phage phiJB (KT344895)
23	NZ_CP012018.1	Gv88	From patient with wound infection	2171984-2220328	48345	Staphylococcus phage SA345ruMSSAST8 (MH401416)

24	NC_021670.1	Bmb9393	N/A	1632359-1696961	64603	Staphylococcus phage P954 (GQ398772)
25	NC_021670.1	Bmb9393	N/A	1165187-1222288	57102	Staphylococcus phage SA780ruMSSAST101 (MH384260)
26	NZ_CP058312.1	BLR-DV	Urine	825844-883738	57895	Staphylococcus phage SPbeta-like 2 (MT955612)
27	NZ_CP058312.1	BLR-DV	Urine	1766550-1829374	62825	Staphylococcus phage phiJB (KT344895)
28	NZ_AP019305.1	TUM9458	N/A	111480-174975	63496	Staphylococcus phage phiETA3 (AP008954)
29	NZ_AP019543.1	KG-18	Blood	1544636-1609705	65070	Staphylococcus phage SA345ruMSSAST8 (MH401416)
30	NZ_AP019543.1	KG-18	Blood	2758048-2816757	58710	Staphylococcus phage SA97 (KJ716334)

Supplementary Table S2. The *Staphylococcus aureus* virulent and temperate phages examined in the study

**No.**	**Phage**	**Family/subfamily**	**Morphology**	**GenBank acc. No.**	**Lifestyle**	**Genome size (bp)**	**Supplementary reference**
1	Sb-1	*Herelleviridae*	~~Myoviridae~~Myovirus-like	NC_023009.1	Virulent	127188	Kvachadze et al. Microb. Biotechnol. 4(5):643–650. (2011)

2	GH15	*Herelleviridae*	~~Myoviridae~~Myovirus-like	NC_019448.1	Virulent	139806	Gu et al. J Clin Microbiol. 49(1):111–117. (2010)
3	SA97	*Azeredovirinae*	~~Siphoviridae~~Siphovirus-like	NC_029010.1	Virulent	40592	Chang et al. Viruses. 7(10):5225–42. (2015)
4	Team1	*Herelleviridae*	~~Myoviridae~~Myovirus-like	NC_025417.1	Virulent	140903	Haddad et al. PLoS One. 9(7): e102600. (2014)
5	K	*Herelleviridae*	~~Myoviridae~~Myovirus-like	NC_005880.2	Virulent	148317	Estrella et al. Bacteriophage. 6(3):e1219440. (2016)
6	P68	*Rountreeviridae*	~~Podoviridae~~Podovirus-like	AF513033.1	Virulent	18227	Haddad et al. Int J Food Microbiol. 18:217:7-13. (2016)
7	phi44AHJD	*Rountreeviridae*	~~Podoviridae~~Podovirus-like	NC_004678.1	Virulent	16784	Haddad et al. Int J Food Microbiol. 18:217:7-13. (2016)
8	vB_SauS_IMEP5	N/A	~~Siphoviridae~~Siphovirus-like	KX156762.1	Virulent	44677	Zhang et.al. Virus Genes. 53(3):464-476. (2017)

9	SLPW	*Rountreeviridae*	~~Podoviridae~~Podovirus-like	NC_031008.1	Virulent	17861	Wang et al. Front Microbiol. 15:7:934. (2016)
10	KMSP1	N/A	~~Myoviridae~~Myovirus-like	ON153212.1	Virulent	138528	Kwak et al. International Journal of Food Microbiology. 2:390:110119. (2023)
11	qdsa002	*Herelleviridae*	~~Myoviridae~~Myovirus-like	KY779849.1	Virulent	142499	Ning et al. Enzyme Microb Technol. 148:109809. (2021)
12	vB_SauM-515A1	*Herelleviridae*	~~Myoviridae~~Myovirus-like	MN047438.1	Virulent	148511	Abdraimova et al. Front. Microbiol. 20:15:1519312. (2024)
13	phiIPLA-RODI	*Herelleviridae*	~~Myoviridae~~Myovirus-like	KP027446.1	Virulent	142348	Gutiérrez et al. Appl Environ Microbiology. 81(10):3336–3348. (2015)

14	vB_SauS_phi2	N/A	~~Siphoviridae~~Siphovirus-like	NC_028862.1	Virulent	44222	Haddad et al. Int J Food Microbiol. 18:217:7-13. (2016)
15	vB_SauM_VL10	*Herelleviridae*	~~Myoviridae~~Myovirus-like	OP940114.1	Virulent	141746	Lerdsittikul et al. Sci Rep. 14(1):9251. (2024)
16	SAPYZU_15	*Herelleviridae*	N/A	MW864252.1	Virulent	135178	Zhou et al. Microbiol Res. 271:127369. (2023)
17	TSP	*Rountreeviridae*	~~Podoviridae~~Podovirus-like	MW286254.1	Virulent	17987	Tabassum et al. Scientific Reports. 12, 10008. (2022)
18	vB_SauR_SW25	*Rountreeviridae*	~~Podoviridae~~Podovirus-like	PP135470.1	Virulent	17223	Banar et al. PLoS One. 20(1):e0316157. (2025)
19	vB_SauR_SW21	*Rountreeviridae*	~~Podoviridae~~Podovirus-like	OR683639.1	Virulent	17369	Banar et al. PLoS One. 20(1):e0316157. (2025)
20	CapO46	*Rountreeviridae*	~~Podoviridae~~Podovirus-like	PV007823.1	Virulent	17107	Cunha et al. Microorganisms. 13(3), 664. (2025)

21	SA46-CTH2	*Rountreeviridae*	~~Podoviridae~~Podovirus-like	MK764384.1	Virulent	17505	Duc et al. Appl Microbiol Biotechnol. 104(11):5145-5158. (2020)
22	CSA13	*Rountreeviridae*	~~Podoviridae~~Podovirus-like	NC_048159.1	Virulent	17034	Cha et al. Food Microbiol. 84:103245. (2019)
23	Stau2	*Herelleviridae*	~~Myoviridae~~Myovirus-like	NC_030933.1	Virulent	133798	Hsieh et al. Appl Environ Microbiol. 77(3):756-61. (2011)
24	IME-SA1	*Herelleviridae*	~~Myoviridae~~Myovirus-like	NC_047729.1	Virulent	140218	Zhang et al. Virol Sin. 30(6):433-440. (2015)
25	IME-SA2	*Herelleviridae*	~~Myoviridae~~Myovirus-like	NC_047730.1	Virulent	140906	Zhang et al. Virol Sin. 30(6):433-440. (2015)
26	IME-SA118	*Herelleviridae*	~~Myoviridae~~Myovirus-like	NC_047731.1	Virulent	139750	Zhang et al. Virol Sin. 30(6):433-440. (2015)
27	IME-SA119	*Herelleviridae*	~~Myoviridae~~Myovirus-like	NC_047732.1	Virulent	141028	Zhang et al. Virol Sin. 30(6):433-440. (2015)

28	vB_SauP_ASUmrsa123	N/A	~~Podoviridae~~Podovirus-like	OR259390.1	Virulent	17155	El-Tawab et al. Virol J. 21:284. (2024)
29	SAPYZU_11	*Rountreeviridae*	~~Podoviridae~~Podovirus-like	MW864250.1	Virulent	17790	Zhou et al. Microbiol Res. 271:127369. (2023)
30	SAML-229	N/A	~~Myoviridae~~Myovirus-like	OP352124.1	Virulent	137842	Brouillette et al. Viruses. 30;15(4):887. (2023)
31	phiETA	*Azeredovirinae*	~~Siphoviridae~~Siphovirus-like	NC_003288.1	Temperate	43081	Yamaguchi et al. Mol Microbiol. 38(4):694-705. (2000)
32	B166	*Azeredovirinae*	~~Siphoviridae~~Siphovirus-like	NC_028859.1	Temperate	42881	Botka et al. Virus Genes. 51(1):122-131. (2015)
33	B236	*Azeredovirinae*	~~Siphoviridae~~Siphovirus-like	NC_028915.1	Temperate	43228	Botka et al. Virus Genes. 51(1):122-131. (2015)
34	phi 13	*Bronfenbrennervirinae*	N/A	AF424783.1	Temperate	42722	Iandolo et al. Gene. 289(1-2):109-18. (2002)
35	phi 12	N/A	N/A	AF424782.1	Temperate	44970	Iandolo et al. Gene. 289(1-2):109-18. (2002)

36	phi 11	*Azeredovirinae*	N/A	NC_004615.1	Temperate	43604	Iandolo et al. Gene. 289(1-2):109-18. (2002)
37	PVL	*Bronfenbrennervirinae*	N/A	AB009866.2	Temperate	41401	Kaneko et al. Biosci Biotechnol Biochem. 61(11):1960-2. (1997)
38	TEM126	*Azeredovirinae*	~~Siphoviridae~~Siphovirus-like	NC_054978.1	Temperate	41882	Lee et al. Arch Virol. 156(4):717-20. (2011)
39	SA75	*Azeredovirinae*	~~Siphoviridae~~Siphovirus-like	NC_054975.1	Temperate	43134	Assad-Garcia et al. Appl Environ Microbiol. 88(3):e0148621. (2022)
40	ASZ22RN	*Azeredovirinae*	~~Siphoviridae~~Siphovirus-like	ON513432.1	Temperate	43579	Kałuski et al. Microbiol Spectr. 13(8):e0333224. (2025)
41	SapYZUs7	N/A	~~Siphoviridae~~Siphovirus-like	OQ828659.1	Temperate	43682	Zhou et al. Microbiol Res. 292:128040. (2025)

42	vB_SauS_321c	*Azeredovirinae*	~~Siphoviridae~~Siphovirus-like	OM439666.1	Temperate	42397	Suárez et al. Microbiol Spectr. 31;10(4):e0033422. (2022)
43	vB_SauS_320	N/A	~~Siphoviridae~~Siphovirus-like	OM439665.1	Temperate	45809	Suárez et al. Microbiol Spectr. 31;10(4):e0033422. (2022)
44	vB_SauS_Mh4	*Azeredovirinae*	~~Siphoviridae~~Siphovirus-like	OM439675.1	Temperate	44199	Suárez et al. Microbiol Spectr. 31;10(4):e0033422. (2022)
45	vB_SauS_760	*Azeredovirinae*	~~Siphoviridae~~Siphovirus-like	OM439669.1	Temperate	42788	Suárez et al. Microbiol Spectr. 31;10(4):e0033422. (2022)
46	vB_SauS_287	*Azeredovirinae*	~~Siphoviridae~~Siphovirus-like	OM439663.1	Temperate	43134	Suárez et al. Microbiol Spectr. 31;10(4):e0033422. (2022)
47	vB_SauS_I73	*Azeredovirinae*	~~Siphoviridae~~Siphovirus-like	OM439672.1	Temperate	43575	Suárez et al. Microbiol Spectr. 31;10(4):e0033422. (2022)

48	vB_SauS_308	*Bronfenbrennervirinae*	~~Siphoviridae~~Siphovirus-like	OM439664.1	Temperate	39968	Suárez et al. Microbiol Spectr. 31;10(4):e0033422. (2022)
49	vB_SauS_690	N/A	~~Siphoviridae~~Siphovirus-like	OM439667.1	Temperate	45771	Suárez et al. Microbiol Spectr. 31;10(4):e0033422. (2022)
50	vB_SauS_713	*Azeredovirinae*	~~Siphoviridae~~Siphovirus-like	OM439668.1	Temperate	42961	Suárez et al. Microbiol Spectr. 31;10(4):e0033422. (2022)
51	vB_SauS_832	*Azeredovirinae*	~~Siphoviridae~~Siphovirus-like	OM439671.1	Temperate	43499	Suárez et al. Microbiol Spectr. 31;10(4):e0033422. (2022)
52	phiMR25	*Azeredovirinae*	N/A	NC_010808.1	Temperate	44342	Hoshiba et al. Arch Virol. 155(4):545-52. (2010)
53	Hesat	*Azeredovirinae*	N/A	OP947204.1	Temperate	43129	Turchi et al. Appl Microbiol Biotechnol. 108(1):299. (2024)

54	PHB21	N/A	~~Siphoviridae~~Siphovirus-like	MW924497.1	Temperate	45260	Yang et al. Virulence. 13(1):137–148. (2022)
55	phinm4	*Azeredovirinae*	N/A	NC_028864.1	Temperate	43189	Bae et al. Mol Microbiol. 62(4):1035-47. (2006)
56	phiNM3	*Bronfenbrennervirinae*	N/A	NC_008617.1	Temperate	44061	Bae et al. Mol Microbiol. 62(4):1035-47. (2006)
57	P240	N/A	N/A	NC_055042.1	Temperate	45985	Kraushaar et al. scientific reports. 7:2004. (2017)
58	P282	*Bronfenbrennervirinae*	N/A	NC_048634.1	Temperate	41960	Kraushaar et al. scientific reports. 7:2004. (2017)
59	P630	*Bronfenbrennervirinae*	N/A	NC_048635.1	Temperate	40448	Kraushaar et al. scientific reports. 7:2004. (2017)
60	P1105	*Bronfenbrennervirinae*	N/A	NC_048636.1	Temperate	42282	Kraushaar et al. scientific reports. 7:2004. (2017)

Supplementary references

1. Kvachadze et al. (2011). Evaluation of lytic activity of staphylococcal bacteriophage Sb-1 against freshly isolated clinical pathogens. Microb. Biotechnol. 4(5):643–650. doi: 10.1111/j.1751-7915.2011.00259.x.
2. Gu et al. (2010). LysGH15, a Novel Bacteriophage Lysin, Protects a Murine Bacteremia Model Efficiently against Lethal Methicillin-Resistant *Staphylococcus aureus* Infection. J Clin Microbiol. 49(1):111–117. doi: 10.1128/JCM.01144-10.
3. Chang et al. (2015). Isolation and Genome Characterization of the Virulent *Staphylococcus aureus* Bacteriophage SA97. Viruses. 7(10):5225–42. doi: 10.3390/v7102870.
4. Haddad et al. (2014). Improving the safety of Staphylococcus aureus polyvalent phages by their production on a *Staphylococcus xylosus* strain. PLoS One. 9(7): e102600. doi: 10.1371/journal.pone.0102600.
5. Estrella et al. (2016). Characterization of novel *Staphylococcus aureus* lytic phage and defining their combinatorial virulence using the OmniLog® system. Bacteriophage. 6(3):e1219440. doi: 10.1080/21597081.2016.1219440.
6. Haddad et al. (2016). Efficacy of two *Staphylococcus aureus* phage cocktails in cheese production. Int J Food Microbiol. 18:217:7-13. doi: 10.1016/j.ijfoodmicro.2015.10.001.
7. Zhang et.al. (2017). Characterization and complete genome sequence analysis of a novel virulent Siphoviridae phage against *Staphylococcus aureus* isolated from bovine mastitis in Xinjiang, China. Virus Genes. 53(3):464-476. doi: 10.1007/s11262-017-1445-z.
8. Wang et al. (2016). SLPW: A Virulent Bacteriophage Targeting Methicillin-Resistant *Staphylococcus aureus* In vitro and In vivo. Front Microbiol. 15:7:934. doi: 10.3389/fmicb.2016.00934.
9. Kwak et al. (2023). Characterization of KMSP1, a newly isolated virulent bacteriophage infecting *Staphylococcus aureus*, and its application to dairy products. Int J Food Microbiol. 2:390:110119. doi: 10.1016/j.ijfoodmicro.2023.110119.
10. Ning et al. (2021). Characterizations of the endolysin Lys84 and its domains from phage qdsa002 with high activities against *Staphylococcus aureus* and its biofilms. Enzyme Microb Technol. 148:109809. doi: 10.1016/j.enzmictec.2021.109809.
11. Abdraimova et al. (2024). Response of *Staphylococcus aureus* to combination of virulent bacteriophage vB_SauM-515A1 and linezolid. Front. Microbiol. 20:15:1519312. doi: 10.3389/fmicb.2024.1519312.
12. Gutiérrez et al. (2015). Two Phages, phiIPLA-RODI and phiIPLA-C1C, Lyse Mono- and Dual-Species Staphylococcal Biofilms. Appl Environ Microbiology. 81(10):3336–3348. doi: 10.1128/AEM.03560-14.
13. Lerdsittikul et al. (2024). Isolation and characterisation of a novel Silviavirus bacteriophage promising antimicrobial agent against methicillin-resistant *Staphylococcus aureus* infections. Sci Rep. 14(1):9251. doi: 10.1038/s41598-024-59903-w.
14. Zhou et al. (2023). WGS analysis of two Staphylococcus aureus bacteriophages from sewage in China provides insights into the genetic feature of highly efficient lytic phages. Microbiol Res. 271:127369. doi: 10.1016/j.micres.2023.127369.

15. Tabassum et al. (2022). TSP, a virulent Podovirus, can control the growth of Staphylococcus aureus for 12 h. Scientific Reports. 12, 10008. https://doi.org/10.1038/s41598-022-13584-5.
16. Banar et al. (2025). A novel broad-spectrum bacteriophage cocktail against methicillin-resistant Staphylococcus aureus: Isolation, characterization, and therapeutic potential in a mastitis mouse model. PLoS One. 20(1):e0316157. doi: 10.1371/journal.pone.0316157.
17. Cunha et al. (2025). Characterization of Newly Isolated Rosenblumvirus Phage Infecting Staphylococcus aureus from Different Sources. Microorganisms. 13(3), 664. https://doi.org/10.3390/microorganisms13030664.
18. Duc et al. (2020). Isolation and application of bacteriophages alone or in combination with nisin against planktonic and biofilm cells of Staphylococcus aureus. Appl Microbiol Biotechnol. 104(11):5145-5158. doi: 10.1007/s00253-020-10581-4.
19. Cha et al. (2019). Effective removal of staphylococcal biofilms on various food contact surfaces by Staphylococcus aureus phage endolysin LysCSA13. Food Microbiol. 84:103245. doi: 10.1016/j.fm.2019.103245.
20. Hsieh et al. (2011). Wide host range and strong lytic activity of Staphylococcus aureus lytic phage Stau2. Appl Environ Microbiol. 77(3):756-61. doi: 10.1128/AEM.01848-10.
21. Zhang et al. (2015). Conserved termini and adjacent variable region of Twortlikevirus Staphylococcus phages. Virol Sin. 30(6):433–440. doi: 10.1007/s12250-015-3643-y.
22. El-Tawab et al. (2024). Characterization and complete genome sequence of highly lytic phage active against methicillin-resistant Staphylococcus aureus (MRSA) isolated from Egypt. Virol J. 21:284. doi: 10.1186/s12985-024-02554-0.
23. Brouillette et al. (2023). Effective Treatment of Staphylococcus aureus Intramammary Infection in a Murine Model Using the Bacteriophage Cocktail StaphLyse™. Viruses. 30;15(4):887. doi: 10.3390/v15040887.
24. Yamaguchi et al. (2000). Phage conversion of exfoliative toxin A production in Staphylococcus aureus. Mol Microbiol. 38(4):694-705. doi: 10.1046/j.1365-2958.2000.02169.x.
25. Botka et al. (2015). Complete genome analysis of two new bacteriophages isolated from impetigo strains of Staphylococcus aureus. Virus Genes. 51(1):122-131. doi: 10.1007/s11262-015-1223-8.
26. Iandolo et al. (2002). Comparative analysis of the genomes of the temperate bacteriophages phi 11, phi 12 and phi 13 of Staphylococcus aureus 8325. Gene. 289(1-2):109-18. doi: 10.1016/s0378-1119(02)00481-x.
27. Kaneko et al. (1997). Panton-valentine leukocidin genes in a phage-like particle isolated from mitomycin C-treated Staphylococcus aureus V8 (ATCC 49775). Biosci Biotechnol Biochem. 61(11):1960-2. doi: 10.1271/bbb.61.1960.
28. Lee et al. (2011). Genomic sequence of temperate phage TEM126 isolated from wild type S. aureus. Arch Virol. 156(4):717-20. doi: 10.1007/s00705-011-0923-1.

29. Assad-Garcia et al. (2022). Cross-Genus “Boot-Up” of Synthetic Bacteriophage in Staphylococcus aureus by Using a New and Efficient DNA Transformation Method. Appl Environ Microbiol. 88(3):e01486-21. doi: 10.1128/AEM.01486-21.
30. Kałuski et al. (2025). Characterization of a novel Phietavirus genus bacteriophage and its potential for efficient transfer of modified shuttle plasmids to Staphylococcus aureus strains of different clonal complexes. Microbiol Spectr. 13(8):e0333224. doi: 10.1128/spectrum.03332-24.
31. Zhou et al. (2025). Temperate bacteriophage SapYZUs7 alters Staphylococcus aureus fitness balance by regulating expression of phage resistance, virulence and antimicrobial resistance gene. Microbiol Res. 292:128040. doi: 10.1016/j.micres.2024.128040.
32. Suárez et al. (2022). Bioinformatic Analysis of a Set of 14 Temperate Bacteriophages Isolated from *Staphylococcus aureus* Strains Highlights Their Massive Genetic Diversity. Microbiol Spectr. 31;10(4):e0033422. doi: 10.1128/spectrum.00334-22.
33. Hoshiba et al. (2010). Isolation and characterization of a novel Staphylococcus aureus bacteriophage, phiMR25, and its therapeutic potential. Arch Virol. 155(4):545-52. doi: 10.1007/s00705-010-0623-2.
34. Turchi et al. (2024). Isolation and characterization of novel Staphylococcus aureus bacteriophage Hesat from dairy origin. Appl Microbiol Biotechnol. 108(1):299. doi: 10.1007/s00253-024-13129-y.
35. Yang et al. (2022). A temperate Siphoviridae bacteriophage isolate from Siberian tiger enhances the virulence of methicillin-resistant Staphylococcus aureus through distinct mechanisms. Virulence. 13(1):137–148. doi: 10.1080/21505594.2021.2022276.
36. Bae et al. (2006). Prophages of Staphylococcus aureus Newman and their contribution to virulence. Mol Microbiol. 62(4):1035-47. doi: 10.1111/j.1365-2958.2006.05441.x.
37. Kraushaar et al. (2017). Acquisition of virulence factors in livestock-associated MRSA: Lysogenic conversion of CC398 strains by virulence gene-containing phages. scientific reports. 7:2004. doi: 10.1038/s41598-017-02175-4.

Supplementary Table S3. Summary statistics of pairwise BLAST-derived DNA identity, genome coverage, and combined genomic distance among the selected *S. aureus* virulent and temperate phages and prophages (valid comparisons only).

Metric	Lifestyle	Mean	Median	SD	Q1-Q3
DNA identity	Virulent	86.89	87.88	9.27	78.85-96.15
	Temperate	83.51	85.35	11.62	75.05–93.88

(%)	Prophage	84.62	86.41	10.48	76.12–93.57
Genome coverage (%)	Virulent	43.90	23.00	44.36	0.50–92.50
	Temperate	38.72	18.50	41.57	1.00–87.75
	Prophage	41.35	21.00	42.88	1.50–89.50
Combined distance	Virulent	0.586	0.793	0.440	0.109–1.014
	Temperate	0.793	0.936	0.401	0.364–1.241
	Prophage	0.735	0.812	0.389	0.322–1.102

Valid comparisons: *n*=308 for the virulent phage genomes; *n*=435 for the temperate phage genomes; *n*=426 for the prophage genomes

Supplementary Table S4. Summary of combined genomic distance metrics among *S. aureus* virulent and temperate phages and prophages (all pairwise comparisons; NSS assigned maximum distance).

**Statistic**	**Virulent**	**Temperate**	**Prophage**
Total pairs	435	435	435
Valid similarity pairs	178 (40.9%)	435 (100%)	411 (94.5%)

NSS pairs	257 (59.1%)	0 (0%)	24 (5.5%)
Mean combined distance	0.942	0.744	0.916
Median combined distance	1.414	0.861	0.846
SD	0.602	0.235	0.276
Q1–Q3	0.135–1.414	0.623-0.923	0.670-0.959
Maximum	1.414	1.404	1.414

Supplementary Table S5. Pairwise PERMANOVA comparisons of gene-level compositional profiles among phage lifestyles

**Group 1**	**Group 2**	**Pseudo-F**	***p***	**R^2**
Virulent	Temperate	818.31	0.001	0.173
Virulent	Prophage	476.36	0.001	0.109
Temperate	Prophage	53.92	0.001	0.014

Supplementary Table S6. Spearman correlation structure of gene-level length and codon usage metrics across phage lifestyles.

Variable pair	Virulent	Temperate	Prophage
CAI vs %GC3	−0.79 [−0.81, −0.77] (<0.001)	−0.76 [−0.79, −0.73] (<0.001)	−0.73 [−0.76, −0.70] (<0.001)
Nc vs %GC3	0.50 [0.47, 0.53] (<0.001)	0.47 [0.43, 0.51] (<0.001)	0.44 [0.40, 0.48] (<0.001)
CAI vs *Nc*	−0.56 [−0.59, −0.53] (<0.001)	−0.52 [−0.56, −0.48] (<0.001)	−0.49 [−0.53, −0.45] (<0.001)
Log_{10}Len vs %GC2	0.38 [0.35, 0.41] (<0.001)	0.34 [0.30, 0.38] (<0.001)	0.31 [0.27, 0.35] (<0.001)
Log_{10}Len vs CAI	−0.09 [−0.12, −0.06] (<0.001)	−0.05 [−0.08, −0.02] (0.008)	−0.04 [−0.07, −0.01] (0.040)
Log_{10}Len vs *Nc*	0.07 [0.04, 0.10] (0.012)	0.11 [0.07, 0.15] (<0.001)	0.08 [0.04, 0.12] (0.020)

Values represent Spearman's ρ with 95% confidence intervals (in brackets), followed by FDR-adjusted *p*-values shown in parentheses.

Supplementary Table S7. Partial Spearman correlations, controlling for %GC3 and overall %GC across phage lifestyles.

Variable pair	Virulent	Temperate	Prophage
Log_{10}Len vs CAI	−0.026 (0.112)	−0.011 (0.384)	−0.008 (0.417)
Log_{10}Len vs *Nc*	0.062 (<0.001)	0.048 (0.021)	0.039 (0.048)
CIA vs *Nc* (controlled)	−0.417 (<0.001)	−0.392 (<0.001)	−0.365 (<0.001)

Values represent partial Spearman correlation coefficients (ρ) controlling for %GC3 and overall %GC, with FDR-adjusted *p*-values shown in parentheses.

Supplementary Table S8. The descriptive *nt* and codon usage statistics determined for the genes across the genome of the *S. aureus* jumbo virulent phage SA1

Metric	Mean	SD	Min	Max	P10	P25	P50	P75	P90	IQR
nt	937.523	1102.681	99	11034	244.2	378.75	640.5	1066	1961.1	687.75
CAI	0.752	0.044	0.456	0.889	0.697	0.728	0.757	0.779	0.799	0.051
%GC	26.52	3.398	18.6	36.8	22.34	24.1	26.4	28.275	30.8	4.175
%GC1	37.521	6.865	18.4	68.8	29.67	33.5	36.8	41.275	45.01	7.775

%GC2	24.391	5.803	11.1	47	17.6	20.4	23.7	28.3	32	7.9
%GC3	17.643	4.462	8.9	57.5	13.3	15.025	16.85	19.375	22.890	4.35
Nc	35.319	4.155	25	56.6	31.3	33	34.6	36.9	39.73	3.9

Supplementary Table S9. The descriptive and comparative *nt* statistics determined across the different functional gene groups of the *S. aureus* virulent and temperate phages, and those of this species prophages

Category	Lifestyle	*n**	Mean	Min	Max	P10	P25	P50	P75	P90	IQR
Structural genes	Virulent	378	1109.19	126	6225	264	456	879	1548	1923	1092
	Temperate	408	1135.82	159	6225	345	414	942	1425	1899	1011
	Prophage	343	1283.95	123	8274	333	495	981	1539	1911	1044
Regulatory genes	Virulent	71	756.55	114	3459	174	220.5	438	741	1944	520.5
	Temperate	190	458.42	144	2448	174	207	331.5	720	792	513
	Prophage	224	562.21	150	2448	174	249	423	765.8	1038	516.8
Lysis genes	Virulent	75	723.4	174	2964	353.4	456	516	804	1469.4	348

	Temperate	62	769.16	135	1875	255	303	438	1446	1455	1143
	Prophage	37	485.11	255	1899	255	255	303	438	1026.6	183
Replication genes	Virulent	112	1356.24	180	3750	459	861	1282.5	1749	2286	888
	Temperate	45	890.33	141	2448	165	771	780	1242	1719	471
	Prophage	21	1435.29	807	1953	894	1242	1380	1953	1953	711
Repair genes	Virulent	126	855	144	1935	378	501	712.5	1036.5	1371	535.5
	Temperate	26	668.77	291	1956	300	315	552	921	1050	606
	Prophage	50	870.66	138	2916	300	321	921	1140	1386	819

n* – A number of genes analyzed

Supplementary Table S10. The descriptive and comparative overall %GC statistics determined across the different functional gene groups of the *S. aureus* virulent and temperate phages, and those of this species prophages

**Category**	**Lifestyle**	***n****	**Mean**	**Min**	**Max**	**P10**	**P25**	**P50**	**P75**	**P90**	**IQR**
	Virulent	378	32.41	23.00	39.50	28.20	30.50	32.30	35.40	36.50	4.90

Structural genes	Temperate	408	34.87	27.50	40.60	31.77	33.48	35.00	36.33	37.70	2.85
	Prophage	343	34.18	27.50	39.30	30.90	32.50	34.50	36.20	36.90	3.70
Regulatory genes	Virulent	71	31.46	24.10	39.80	27.50	28.15	31.50	33.95	34.90	5.80
	Temperate	190	34.76	23.30	43.70	30.56	32.60	35.20	37.30	39.22	4.70
	Prophage	224	34.10	23.30	41.40	29.70	31.85	34.40	36.50	38.48	4.65
Lysis genes	Virulent	75	33.96	25.50	41.40	26.58	33.85	35.10	36.35	38.16	2.50
	Temperate	62	36.33	30.70	41.50	32.32	33.70	37.00	38.88	40.56	5.18
	Prophage	37	34.57	30.50	40.80	32.12	32.50	32.80	37.00	38.60	4.50
Replication genes	Virulent	112	30.42	23.30	35.00	28.20	29.10	30.40	31.20	32.90	2.10
	Temperate	45	34.02	26.50	39.60	31.54	33.10	34.20	35.50	36.70	2.40
	Prophage	21	33.84	26.50	36.10	32.50	33.70	33.70	35.00	36.00	1.30
Repair genes	Virulent	126	30.15	24.50	36.80	27.40	27.90	29.50	32.50	34.40	4.60

	Temperate	26	30.82	26.60	35.30	28.25	29.55	30.55	31.93	33.70	2.38
	Prophage	50	31.97	24.10	41.60	27.79	30.30	32.10	33.88	34.72	3.58

n* – A number of genes analyzed

Supplementary Table S11. The descriptive and comparative %GC1 statistics determined across the different functional gene groups of the *S. aureus* virulent and temperate phages, and those of this species prophages

**Category**	**Lifestyle**	***n****	**Mean**	**Min**	**Max**	**P10**	**P25**	**P50**	**P75**	**P90**	**IQR**
Structural genes	Virulent	378	44.16	15.20	57.30	37.10	40.65	44.30	48.30	50.90	7.65
	Temperate	408	45.72	36.60	55.90	41.57	43.50	45.30	47.60	51.30	4.10
	Prophage	343	45.05	35.70	55.90	41.22	43.30	44.70	47.20	50.20	3.90
Regulatory genes	Virulent	71	43.19	33.30	59.00	35.60	39.60	44.10	45.60	48.70	6.00
	Temperate	190	44.46	29.50	54.10	36.88	41.70	45.30	47.58	50.82	5.88
	Prophage	224	44.44	22.40	57.30	38.40	40.70	45.00	48.00	50.60	7.30
Lysis genes	Virulent	75	44.92	33.30	54.20	35.66	42.70	45.90	47.35	54.20	4.65

	Temperate	62	42.88	31.70	50.00	38.70	40.00	43.00	45.08	48.77	5.08
	Prophage	37	41.85	37.80	50.00	38.60	40.00	40.00	45.20	45.48	5.20
Replication genes	Virulent	112	42.64	26.70	57.40	36.01	38.20	43.60	44.90	46.88	6.70
	Temperate	45	43.13	27.70	54.70	38.26	39.30	43.10	45.80	47.52	6.50
	Prophage	21	44.03	37.60	47.60	38.60	43.00	43.90	47.60	47.60	4.60
Repair genes	Virulent	126	41.62	28.00	51.60	32.20	35.75	43.10	47.60	49.40	11.85
	Temperate	26	39.83	34.10	48.80	37.00	37.10	38.60	41.10	47.75	4.00
	Prophage	50	41.90	32.50	51.90	37.10	38.00	39.10	47.65	50.27	9.65

n* – A number of genes analyzed

Supplementary Table S12. The descriptive and comparative %GC2 statistics determined across the different functional gene groups of the *S. aureus* virulent and temperate phages, and those of this species prophages

**Category**	**Lifestyle**	***n****	**Mean**	**Min**	**Max**	**P10**	**P25**	**P50**	**P75**	**P90**	**IQR**
Structural genes	Virulent	378	33.08	16.70	48.40	24.00	29.20	33.10	37.60	40.73	8.40
	Temperate	408	32.13	17.40	42.80	26.30	29.30	31.90	34.93	39.00	5.63

	Prophage	343	31.52	17.40	41.20	25.50	29.30	32.00	34.20	38.70	4.90
Regulatory genes	Virulent	71	28.86	15.40	39.50	22.40	24.90	27.60	33.45	37.00	8.55
	Temperate	190	29.61	16.70	39.70	24.10	26.00	29.95	33.30	34.80	7.30
	Prophage	224	29.76	17.30	38.90	25.40	26.50	30.20	32.40	34.51	5.90
Lysis genes	Virulent	75	35.77	20.70	47.10	23.60	32.50	37.00	41.40	43.80	8.90
	Temperate	62	37.72	26.70	45.50	32.90	34.03	38.10	40.05	42.48	6.03
	Prophage	37	35.67	31.40	42.50	32.90	32.90	33.70	39.70	39.70	6.80
Replication genes	Virulent	112	27.48	22.40	34.70	24.31	26.30	27.90	28.50	29.59	2.20
	Temperate	45	30.11	20.00	41.40	24.50	29.00	30.80	31.90	34.40	2.90
	Prophage	21	30.98	23.50	34.70	29.00	29.20	30.90	34.60	34.60	5.40
Repair genes	Virulent	126	29.90	20.90	43.30	23.65	24.50	28.35	33.50	40.00	9.00
	Temperate	26	27.03	22.90	34.20	23.20	24.50	26.70	28.23	30.40	3.73

	Prophage	50	28.77	21.50	50.90	23.50	26.20	28.00	30.30	32.34	4.10

n* – A number of genes analyzed

Supplementary Table S13. The descriptive and comparative %GC3 statistics determined across the different functional gene groups of the *S. aureus* virulent and temperate phages, and those of this species prophages

Category	Lifestyle	*n**	Mean	Min	Max	P10	P25	P50	P75	P90	IQR
Structural genes	Virulent	378	19.98	9.20	51.90	14.30	17.23	19.80	22.48	25.90	5.25
	Temperate	408	26.75	16.60	41.30	21.04	24.08	26.90	29.10	32.50	5.03
	Prophage	343	25.97	17.00	39.00	20.40	23.15	25.90	28.90	30.80	5.75
Regulatory genes	Virulent	71	22.30	13.70	48.30	17.10	18.40	21.50	24.90	29.50	6.50
	Temperate	190	30.22	15.00	52.20	20.50	24.85	29.40	34.88	39.62	10.03
	Prophage	224	28.12	15.00	48.30	18.50	22.70	26.95	32.35	37.30	9.65
Lysis genes	Virulent	75	21.21	14.30	40.90	16.10	18.45	19.90	22.10	28.62	3.65
	Temperate	62	28.42	20.40	37.00	23.14	24.73	28.25	31.30	33.68	6.58

	Prophage	37	26.21	16.80	35.90	22.80	24.70	24.80	26.70	30.80	2.00
Replication genes	Virulent	112	21.15	16.40	29.70	18.80	19.90	20.80	22.48	24.10	2.58
	Temperate	45	28.80	18.30	39.60	22.06	23.10	29.20	33.50	35.80	10.40
	Prophage	21	26.49	18.30	36.00	22.90	23.00	26.10	27.20	36.00	4.20
Repair genes	Virulent	126	18.93	10.40	31.10	13.50	17.30	19.55	20.88	23.30	3.58
	Temperate	26	25.57	18.30	31.90	21.75	23.80	25.50	27.68	30.00	3.88
	Prophage	50	25.26	14.50	32.80	18.30	23.10	24.60	28.60	30.06	5.50

n* – A number of genes analyzed

Supplementary Table S14. The descriptive and comparative *Nc* statistics determined across the different functional gene groups of the *S. aureus* virulent and temperate phages, and those of this species prophages

**Category**	**Lifestyle**	***n****	**Mean**	**Min**	**Max**	**P10**	**P25**	**P50**	**P75**	**P90**	**IQR**
Structural genes	Virulent	378	36.75	26.00	50.60	32.90	34.45	36.40	38.30	42.30	3.85
	Temperate	408	44.04	29.10	61.00	39.20	41.38	43.55	46.10	49.32	4.73

	Prophage	343	43.38	30.20	61.00	37.00	40.50	43.20	44.70	49.00	4.20
Regulatory genes	Virulent	71	38.73	28.70	61.00	32.90	34.20	37.20	41.80	45.60	7.60
	Temperate	190	45.01	28.20	61.00	35.89	39.30	43.50	48.48	60.19	9.18
	Prophage	224	44.91	28.10	61.00	37.10	40.80	43.35	47.63	61.00	6.83
Lysis genes	Virulent	75	38.88	30.10	57.60	34.14	36.15	38.30	40.50	42.94	4.35
	Temperate	62	42.08	33.90	47.70	37.40	40.20	41.80	45.28	46.60	5.08
	Prophage	37	39.25	33.90	47.70	34.40	37.40	37.40	41.60	43.10	4.20
Replication genes	Virulent	112	37.81	31.90	46.10	34.30	35.90	38.10	39.03	40.39	3.13
	Temperate	45	46.12	31.90	61.00	39.08	41.80	46.80	49.30	51.50	7.50
	Prophage	21	44.38	35.80	51.10	40.30	41.70	45.40	46.40	50.60	4.70
Repair genes	Virulent	126	36.87	28.80	47.60	33.35	35.10	36.70	38.80	40.70	3.70
	Temperate	26	39.93	31.90	45.80	34.75	37.65	40.85	43.23	43.85	5.58

Prophage	50	40.88	31.30	49.30	35.54	36.58	41.60	42.90	47.10	6.33

n* – A number of genes analyzed

Supplementary Table S15. The descriptive and comparative CAI statistics determined across the different functional gene groups of the *S. aureus* virulent and temperate phages, and those of this species prophages

Category	Lifestyle	*n**	Mean	Min	Max	P10	P25	P50	P75	P90	IQR
Structural genes	Virulent	378	0.73	0.49	0.83	0.67	0.70	0.72	0.75	0.78	0.05
	Temperate	408	0.67	0.57	0.80	0.63	0.65	0.67	0.69	0.72	0.04
	Prophage	343	0.68	0.56	0.78	0.62	0.66	0.68	0.71	0.74	0.05
Regulatory genes	Virulent	71	0.71	0.53	0.83	0.64	0.68	0.73	0.75	0.76	0.07
	Temperate	190	0.64	0.46	0.80	0.55	0.59	0.64	0.68	0.72	0.09
	Prophage	224	0.66	0.53	0.80	0.56	0.61	0.66	0.72	0.75	0.10
Lysis genes		75	0.73	0.53	0.81	0.67	0.72	0.73	0.75	0.78	0.04

	Virulent										
	Temperate	62	0.67	0.60	0.74	0.62	0.64	0.66	0.70	0.70	0.06
	Prophage	37	0.69	0.61	0.79	0.64	0.68	0.70	0.70	0.72	0.03
Replication genes	Virulent	112	0.72	0.65	0.77	0.67	0.71	0.72	0.73	0.75	0.02
	Temperate	45	0.65	0.54	0.77	0.58	0.61	0.64	0.69	0.70	0.08
	Prophage	21	0.67	0.58	0.74	0.58	0.66	0.68	0.70	0.70	0.04
Repair genes	Virulent	126	0.73	0.61	0.84	0.68	0.70	0.72	0.76	0.80	0.06
	Temperate	26	0.70	0.62	0.78	0.65	0.66	0.69	0.73	0.74	0.07
	Prophage	50	0.69	0.61	0.77	0.63	0.65	0.68	0.73	0.76	0.08

n* – A number of genes analyzed

Supplementary Table S16. Pairwise comparisons among the functional gene categories for *nt*, overall GC and codon-positioned GC content, CAI, and *Nc* within phage lifestyles, showing pooled Cliff's δ effect sizes and Holm-adjusted *p*-values.

Metric	Functional Contrast	Direction (median)	Cliff's δ (pooled)	Holm p (pooled)	Also significant within lifestyle(s)
CAI	Replication vs Structural	Replication < Structural	-0.103	0.0001	Pooled only

CAI	Lysis vs Regulatory	Lysis > Regulatory	0.278	0.0008	Pooled only
CAI	Replication vs Repair	Replication < Repair	-0.132	0.004	Temperate
CAI	Structural vs Regulatory	Structural > Regulatory	0.296	0.0135	Temperate
CAI	Lysis vs Replication	Lysis > Replication	0.156	3.10E-06	Virulent, Temperate
GC	Lysis vs Replication	Lysis > Replication	0.423	0.0004	Virulent
GC	Replication vs Repair	Replication > Repair	0.296	0.0005	Temperate, Prophage
GC	Replication vs Structural	Replication < Structural	-0.268	0.0355	Pooled only
GC	Replication vs Regulatory	Replication < Regulatory	-0.304	0.0474	Pooled only
GC	Lysis vs Repair	Lysis > Repair	0.728	2.22E-15	Virulent, Temperate, Prophage
GC	Regulatory vs Repair	Regulatory > Repair	0.653	2.62E-10	Temperate, Prophage
GC	Structural vs Repair	Structural > Repair	0.632	4.42E-11	Virulent, Temperate, Prophage
GC1	Lysis vs Repair	Lysis > Repair	0.42	0.0007	Temperate
GC1	Replication vs Regulatory	Replication < Regulatory	-0.335	0.0038	Pooled only
GC1	Lysis vs Regulatory	Lysis < Regulatory	-0.254	0.0187	Pooled only

GC1	Replication vs Structural	Replication < Structural	-0.305	0.0187	Pooled only
GC1	Replication vs Repair	Replication > Repair	0.272	0.0187	Temperate, Prophage
GC1	Structural vs Repair	Structural > Repair	0.528	1.35E-08	Temperate, Prophage
GC1	Regulatory vs Repair	Regulatory > Repair	0.592	9.87E-10	Temperate, Prophage
GC2	Lysis vs Replication	Lysis > Replication	0.89	0.00E+00	Virulent, Temperate, Prophage
GC2	Lysis vs Regulatory	Lysis > Regulatory	0.866	0.00E+00	Pooled only
GC2	Lysis vs Repair	Lysis > Repair	0.915	0.00E+00	Virulent, Temperate, Prophage
GC2	Structural vs Regulatory	Structural > Regulatory	0.534	2.99E-07	Prophage
GC2	Lysis vs Structural	Lysis > Structural	0.677	6.04E-07	Pooled only
GC2	Replication vs Structural	Replication < Structural	-0.672	7.16E-09	Pooled only
GC2	Structural vs Repair	Structural > Repair	0.796	9.25E-12	Temperate, Prophage
GC3	Replication vs Repair	Replication > Repair	0.12	0.0002	Virulent, Temperate
GC3	Structural vs Regulatory	Structural < Regulatory	-0.321	0.0024	Temperate
GC3	Lysis vs Replication	Lysis < Replication	-0.025	0.0056	Virulent
GC3	Regulatory vs Repair	Regulatory > Repair	0.299	0.0056	Temperate

GC3	Replication vs Structural	Replication > Structural	0.137	6.39E-05	Pooled only
Length_nt	Replication vs Repair	Replication > Repair	0.529	0.0004	Virulent, Prophage
Length_nt	Regulatory vs Repair	Regulatory < Repair	-0.53	0.0007	Prophage
Length_nt	Lysis vs Repair	Lysis < Repair	-0.324	0.0073	Prophage
Length_nt	Structural vs Regulatory	Structural > Regulatory	0.777	0.00E+00	Temperate, Prophage
Length_nt	Replication vs Regulatory	Replication > Regulatory	0.674	1.07E-14	Pooled only
Length_nt	Lysis vs Replication	Lysis < Replication	-0.658	1.15E-12	Virulent, Prophage
Length_nt	Lysis vs Structural	Lysis < Structural	-0.738	2.00E-15	Pooled only
Length_nt	Structural vs Repair	Structural > Repair	0.537	5.97E-05	Temperate
Nc	Replication vs Structural	Replication > Structural	0.13	0.0003	Pooled only
Nc	Regulatory vs Repair	Regulatory > Repair	0.289	0.0182	Temperate
Nc	Lysis vs Regulatory	Lysis < Regulatory	-0.321	0.0222	Pooled only
Nc	Replication vs Regulatory	Replication < Regulatory	-0.005	0.0234	Pooled only
Nc	Lysis vs Replication	Lysis < Replication	-0.282	1.81E-07	Temperate, Prophage

Nc	Replication vs Repair	Replication > Repair	0.283	3.21E-07	Temperate

Pooled Cliff's δ values represent effect sizes calculated across all genomes combined. Pooled *p*-values were Holm-adjusted. The lifestyle column indicates in which individual lifestyle subsets (virulent, temperate, prophage) the same contrast remained statistically significant after independent Holm correction. "Pooled only" denotes contrasts significant in the combined dataset but not within any single lifestyle subset.

Supplementary Table S17. Significant pairwise lifestyle contrasts (virulent, temperate, prophage) within functional gene groups for *nt* and codon usage metrics, with Cliff's δ effect sizes and Holm-adjusted *p*-values.

Metric	Functional Group	Lifestyle Comparison	Direction	Cliffs delta	Holm p
CAI	Lysis	Virulent vs Temperate	Virulent > Temperate	0.8	3.03E-07
CAI	Lysis	Virulent vs Prophage	Virulent > Prophage	0.748	1.26E-06
CAI	Lysis	Temperate vs Prophage	Temperate < Prophage	-0.397	0.00838
GC	Lysis	Temperate vs Prophage	Temperate > Prophage	0.478	0.004367
GC1	Lysis	Virulent vs Prophage	Virulent > Prophage	0.36	0.048943
GC1	Lysis	Temperate vs Prophage	Temperate > Prophage	0.356	0.048943
GC2	Lysis	Temperate vs Prophage	Temperate > Prophage	0.48	0.003972

GC3	Lysis	Virulent vs Temperate	Virulent < Temperate	-0.794	3.82E-07
GC3	Lysis	Virulent vs Prophage	Virulent < Prophage	-0.73	2.32E-06
GC3	Lysis	Temperate vs Prophage	Temperate > Prophage	0.318	0.034519
nt	Lysis	Virulent vs Prophage	Virulent > Prophage	0.713	5.73E-06
nt	Lysis	Temperate vs Prophage	Temperate > Prophage	0.627	5.23E-05
Nc	Lysis	Virulent vs Temperate	Virulent < Temperate	-0.564	0.000355
Nc	Lysis	Temperate vs Prophage	Temperate > Prophage	0.607	0.000159
CAI	Regulatory	Virulent vs Temperate	Virulent > Temperate	0.873	6.61E-07
CAI	Regulatory	Virulent vs Prophage	Virulent > Prophage	0.823	2.07E-06
CAI	Regulatory	Temperate vs Prophage	Temperate < Prophage	-0.339	0.024599
GC	Regulatory	Virulent vs Temperate	Virulent < Temperate	-0.83	2.54E-06
GC	Regulatory	Virulent vs Prophage	Virulent < Prophage	-0.692	8.18E-05
GC	Regulatory	Temperate vs Prophage	Temperate > Prophage	0.314	0.037029
GC3	Regulatory	Virulent vs Temperate	Virulent < Temperate	-0.862	9.48E-07

GC3	Regulatory	Virulent vs Prophage	Virulent < Prophage	-0.817	2.53E-06
GC3	Regulatory	Temperate vs Prophage	Temperate > Prophage	0.372	0.013528
Nc	Regulatory	Virulent vs Temperate	Virulent < Temperate	-0.822	3.10E-06
Nc	Regulatory	Virulent vs Prophage	Virulent < Prophage	-0.823	3.10E-06
CAI	Repair	Virulent vs Temperate	Virulent > Temperate	0.474	0.042137
CAI	Repair	Virulent vs Prophage	Virulent > Prophage	0.692	0.000489
GC	Repair	Virulent vs Prophage	Virulent < Prophage	-0.719	0.000263
GC1	Repair	Virulent vs Temperate	Virulent > Temperate	0.54	0.02553
GC2	Repair	Virulent vs Temperate	Virulent > Temperate	0.647	0.004792
GC2	Repair	Temperate vs Prophage	Temperate < Prophage	-0.477	0.016107
GC3	Repair	Virulent vs Temperate	Virulent < Temperate	-0.904	1.96E-05
GC3	Repair	Virulent vs Prophage	Virulent < Prophage	-0.942	8.16E-07
nt	Repair	Temperate vs Prophage	Temperate < Prophage	-0.496	0.017355
Nc	Repair	Virulent vs Temperate	Virulent < Temperate	-0.765	0.000379
Nc	Repair	Virulent vs Prophage	Virulent < Prophage	-0.828	1.90E-05

CAI	Replication	Virulent vs Temperate	Virulent > Temperate	0.934	4.54E-08
CAI	Replication	Virulent vs Prophage	Virulent > Prophage	0.947	2.53E-06
GC	Replication	Virulent vs Temperate	Virulent < Temperate	-0.908	1.13E-07
GC	Replication	Virulent vs Prophage	Virulent < Prophage	-0.873	1.58E-05
GC2	Replication	Virulent vs Temperate	Virulent < Temperate	-0.574	0.001007
GC2	Replication	Virulent vs Prophage	Virulent < Prophage	-0.825	7.25E-05
GC3	Replication	Virulent vs Temperate	Virulent < Temperate	-0.821	1.96E-06
GC3	Replication	Virulent vs Prophage	Virulent < Prophage	-0.711	0.000557
nt	Replication	Virulent vs Temperate	Virulent > Temperate	0.762	1.14E-05
nt	Replication	Temperate vs Prophage	Temperate < Prophage	-0.762	0.000401
Nc	Replication	Virulent vs Temperate	Virulent < Temperate	-0.939	3.84E-08
Nc	Replication	Virulent vs Prophage	Virulent < Prophage	-0.942	2.88E-06
CAI	Structural	Virulent vs Temperate	Virulent > Temperate	0.922	2.65E-09
CAI	Structural	Virulent vs Prophage	Virulent > Prophage	0.862	2.00E-08

GC	Structural	Virulent vs Temperate	Virulent < Temperate	-0.751	1.80E-06
GC	Structural	Virulent vs Prophage	Virulent < Prophage	-0.627	6.27E-05
GC3	Structural	Virulent vs Temperate	Virulent < Temperate	-0.932	1.74E-09
GC3	Structural	Virulent vs Prophage	Virulent < Prophage	-0.873	1.29E-08
Nc	Structural	Virulent vs Temperate	Virulent < Temperate	-0.95	8.16E-10
Nc	Structural	Virulent vs Prophage	Virulent < Prophage	-0.936	1.00E-09

Pairwise lifestyle differences within each functional gene group. For each functional category and metric, significant pairwise comparisons among the genes of the virulent and temperate phages, and those of the prophages are shown. Effect sizes are reported as Cliff's delta (δ), with direction indicating which lifestyle exhibits higher values. *P*-values are Holm-adjusted. Only contrasts with adjusted $p < 0.05$ are included.

Supplementary Table S18. The descriptive and comparative *nt* statistics determined across the different functional gene groups of the *S. aureus* jumbo virulent phage SA1.

**Gene category**	***n****	**Mean**	**Min**	**Max**	**P10**	**P25**	**P50**	**P75**	**P90**	**IQR**
Structural genes	15	2210.4	408	6063	729	840	1524	2343	5936.4	1503
Lysis genes	1	837	837	837	N/A	N/A	N/A	N/A	N/A	N/A

Replication genes	4	1834	1053	4257	1235.4	1461.75	1671	1926	2117.7	464.25
Repair genes	12	1089.75	417	1980	489.9	599.25	981	1471.5	1776.6	872.25

n* – A number of genes analyzed

Supplementary Table S19. The descriptive and comparative overall %GC statistics determined across the different functional gene groups of the *S. aureus* jumbo virulent phage SA1.

Gene category	Mean	Min	Max	P10	P25	P50	P75	P90	IQR
Structural genes	28.91	25.30	35.00	25.70	26.90	29.10	30.35	31.64	3.45
Lysis genes	36.20	36.20	36.20	N/A	N/A	N/A	N/A	N/A	N/A
Replication genes	27.58	23.10	29.00	26.82	27.08	28.00	28.60	28.90	1.53
Repair genes	28.05	26.10	29.80	26.70	27.60	28.15	28.60	29.32	1.00

Supplementary Table S20. The descriptive and comparative %GC1 statistics determined across the different functional gene groups of the *S. aureus* jumbo virulent phage SA1.

Gene category	Mean	Min	Max	P10	P25	P50	P75	P90	IQR
Structural genes	39.35	32.30	49.90	33.50	36.75	39.40	41.25	43.48	4.50
Lysis genes	41.60	41.60	41.60	N/A	N/A	N/A	N/A	N/A	N/A
Replication genes	38.73	30.20	42.20	35.52	38.18	39.55	40.62	41.06	2.45
Repair genes	38.28	33.80	42.40	34.01	34.33	38.45	42.40	42.40	8.08

Supplementary Table S21. The descriptive and comparative %GC2 statistics determined across the different functional gene groups of the *S. aureus* jumbo virulent phage SA1.

Gene category	Mean	Min	Max	P10	P25	P50	P75	P90	IQR
Structural genes	30.99	24.30	38.30	27.00	27.80	30.20	34.65	36.26	6.85
Lysis genes	47.00	47.00	47.00	N/A	N/A	N/A	N/A	N/A	N/A
Replication genes	26.85	22.80	32.00	24.01	25.30	27.20	27.82	28.80	2.52
Repair genes	29.18	25.50	32.40	25.89	26.48	29.40	32.10	32.28	5.62

Supplementary Table S22. The descriptive and comparative %GC3 statistics determined across the different functional gene groups of the *S. aureus* jumbo virulent phage SA1.

Gene category	Mean	Min	Max	P10	P25	P50	P75	P90	IQR
Structural genes	16.42	12.00	19.40	13.54	14.85	16.20	18.90	19.02	4.05
Lysis genes	20.10	20.10	20.10	N/A	N/A	N/A	N/A	N/A	N/A

Replication genes	17.17	15.70	19.80	15.83	16.18	16.60	17.88	19.33	1.70
Repair genes	16.62	15.00	18.20	15.09	15.22	16.65	18.05	18.14	2.82

Supplementary Table S23. The descriptive and comparative CAI statistics determined across the different functional gene groups of the *S. aureus* jumbo virulent phage SA1.

**Gene category**	**Mean**	**Min**	**Max**	**P10**	**P25**	**P50**	**P75**	**P90**	**IQR**
Structural genes	0.76	0.68	0.80	0.73	0.74	0.75	0.79	0.80	0.04
Lysis genes	0.73	0.73	0.73	N/A	N/A	N/A	N/A	N/A	N/A
Replication genes	0.76	0.71	0.78	0.74	0.75	0.76	0.77	0.77	0.02
Repair genes	0.76	0.72	0.80	0.73	0.75	0.77	0.78	0.79	0.04

Supplementary Table S24. The descriptive and comparative *Nc* statistics determined across the different functional gene groups of the *S. aureus* jumbo virulent phage SA1.

**Gene category**	**Mean**	**Min**	**Max**	**P10**	**P25**	**P50**	**P75**	**P90**	**IQR**
Structural genes	34.21	31.20	37.80	32.24	32.95	34.10	35.10	36.86	2.15
Lysis genes	32.40	32.40	32.40	N/A	N/A	N/A	N/A	N/A	N/A
Replication genes	35.24	32.80	38.90	33.90	33.98	34.80	36.52	36.60	2.55
Repair genes	33.62	32.60	34.60	32.75	32.98	33.65	34.30	34.48	1.33
